# Deciphering the Molecular Mechanisms of Chilling Tolerance in *Lsi1*-Overexpressing Rice

**DOI:** 10.3390/ijms23094667

**Published:** 2022-04-23

**Authors:** Zhong Li, Muhammad Umar Khan, Xue Yan, Dan Mu, Yuebin Xie, Muhammad Waqas, Xin Wu, Puleng Letuma, Changxun Fang, Wenxiong Lin

**Affiliations:** 1 Fujian Provincial Key Laboratory of Agroecological Processing and Safety Monitoring, College of Life Sciences, Fujian Agriculture and Forestry University, Fuzhou 350002, China; lizhong021@126.com (Z.L.); yanxue@fafu.edu.cn (X.Y.); mudan@fafu.edu.cn (D.M.); 13426544042@163.com (Y.X.); waqasjutt_19@yahoo.com (M.W.); wendyxxxi@163.com (X.W.); 2 Key Laboratory of Crop Ecology and Molecular Physiology, Fujian Agriculture and Forestry University, Fuzhou 350002, China; umar.khan018@yahoo.com; 3 Crop Science Department, Faculty of Agriculture, National University of Lesotho, Maseru 100, Lesotho; pulengletuma@yahoo.com

**Keywords:** rice, silicon, low temperature sensitization, *Lsi1* gene overexpression, proteomics, interacting proteins

## Abstract

Improving tolerance to low-temperature stress during the rice seedling stage is of great significance in agricultural science. In this study, using the low silicon gene 1 (*Lsi1*)-overexpressing (Dular-OE) and wild-type rice (Dular-WT), we showed that *Lsi1* overexpression enhances chilling tolerance in Dular-OE. The overexpression of the *Lsi1* increases silicon absorption, but it was not the main reason for chilling tolerance in Dular-OE. Instead, our data suggest that the overexpression of a *Lsi1*-encoding NIP and its interaction with key proteins lead to chilling tolerance in Dular-OE. Additionally, we show that the high-mobility group protein (HMG1) binds to the promoter of *Lsi1*, positively regulating its expression. Moreover, Nod26-like major intrinsic protein (NIP)’s interaction with α and β subunits of ATP synthase and the 14-3-3f protein was validated by co-immunoprecipitation (Co-IP), bimolecular fluorescent complementary (BiFC), and GST-pulldown assays. Western blotting revealed that the overexpression of NIP positively regulates the ATP-synthase subunits that subsequently upregulate calcineurin B-like interacting protein kinases (CIPK) negatively regulating 14-3-3f. Overall, these NIP-mediated changes trigger corresponding pathways in an orderly manner, enhancing chilling tolerance in Dular-OE.

## 1. Introduction

Crop plants encounter various environmental stresses during their life cycles. Low-temperature stress, which can be categorized as chilling (0–15 °C) and freezing (<0 °C) stress, is a leading climatic factor limiting plant growth, productivity, and the geographic distributions of crop plants [1]. In China, the frequent temperature drop in late spring is an inevitable climate phenomenon, seriously hampering both the seed germination and the seedling growth of rice (*Oryza sativa*). This sudden temperature drop in a short period has a devastating impact, resulting in the inability of 30–50% of seedlings to grow due to them rotting, and this reduces rice yields by 3–5 billion kilograms per year [2]. The city of Fuzhou in China faces temperature drops below 15 °C during the months of March to April (http://www.meteomanz.com/sy3?l=1&cou=2250&ind=58847&m1=03&y1=2021&m2=04&y2=2021) (accessed on 15 April 2022), resulting in a decrease of yield. Rice (*Oryza sativa* L.) is a staple food for half of the world’s population and is extensively grown by more than half of the world’s farmers [3,4]. Rice is particularly sensitive to low-temperature stress due to its tropical and subtropical origin. In temperate areas, rice production can be devastated by low-temperature stress [5]. Therefore, the development of cold-tolerant rice varieties is urgently needed. Several studies have found that low temperatures significantly decrease the chlorophyll contents, photosynthesis rate, growth index, and physiological enzyme activity of rice [2,6,7]. However, rice pretreated with a low temperature before actual exposure to low-temperature stress showed a reduced effect, indicating that the chilling tolerance could be induced in rice [8].

Rice has developed adaptive strategies to cope with low-temperature stress. Elements such as silicon (Si) play a significant role in strengthening the crop plant’s defense mechanisms. The addition of Si can decrease lipid peroxidation and membrane permeability and an increase in the antioxidant defense activity in wheat and cucumbers [9,10]. In rice, silicon absorption by the root system involves an aquaporin protein (Nod26-like major intrinsic protein; NIP) encoded by the low silicon gene 1 (*Lsi1*). NIP is mainly located in the root’s outer cortex and the outer membrane of the inner cortex, where the Casparian strips are located. Additionally, expression of *Lsi1* in *Xenopus* oocytes was linked to silicon transportation activity, indicating that the *Lsi1* encodes a silicon transporter [11]. Lemont and Dular, two rice varieties, show significant differences in their resistance to ultraviolet B (UV-B) radiation. The results show that the difference is due to differences in silicon content and *Lsi1* gene expression, which are significantly higher in Lemont than in the UV-B-sensitive Dular rice [12]. Moreover, RNA interference (RNAi) and overexpression studies in Lemont rice revealed that *Lsi1* gene expression was positively correlated with UV-B radiation resistance. Based on the Coding sequence (CDS) of rice *Lsi1*, our research group designed the PCR primers to amplify rice *Lsi1* and inserted them into a gene-overexpression vector Ubiquitin 1301 to construct the recombinant plasmid (*Lsi1*-Ubiquitin1301), and then transformed it into Agrobacterium EHA105; using Agrobacterium-mediated genetic transformation system, we obtained the Dular’s mutants with *Lsi1* overexpression (Dular-OE). Previously, we showed that wild-type Dular rice with a low content of silicon in the leaves, suffered significant chilling injury at temperatures < 15 °C, while transgenic Dular overexpressing *Lsi1* (Dular-OE) was resistant [7]. This stimulated our interest in further study.

Previously, RNA-seq technology revealed several differentially expressed genes between Dular-WT and Dular-OE seedlings. Importantly, in Dular-OE, genes related to photosynthesis were upregulated, while the genes encoding the proteasome were downregulated when Dular-OE seedlings were exposed to low temperatures. Additionally, at low temperatures, serine hydroxymethyltransferase (SHMT), peptidyl-prolyl cis-trans isomerase (PPIase), and bifunctional 3′-phoshoadenosine 5′-phosphosulfate synthetase (PAPSS) were upregulated in Dular-OE plants, while these were downregulated in Dular-WT [2]. Notably, the OsSHMT protein is known to interact with the α and β subunits of ATP synthase, heat shock protein (HSP), mitochondrial substrate carrier family protein E (MSCP), and ascorbate peroxidase (APX), promoting the elimination of reactive oxygen species (ROS) [13] and has been linked to improved chilling tolerance in rice. However, these findings only provide indirect insights into *Lsi1*-mediated chilling tolerance in rice. The underlying molecular mechanism of the chilling sensitivity in Dular-WT compared to Dular-OE is still unclear.

One study about Dular’s sensitivity to low temperature identified the role of an RNA-binding protein, DUA1. It has a high sequence homology with the pentapeptide repeat family and participates in plastid RNA editing at low temperatures. Moreover, DUA1 can transcriptionally bind to the ribosomal protein rps8-182. The study further showed that the DUA1 gene was located in an 11KB region on chromosome 9, and more importantly, it had eight base deletions in Dular compared to Nipponbare. This is a transcoding mutation that impairs the RNA editing of *DUA1* in Dular. The RNA-editing cofactor WSP1, involved in chloroplast development at low temperatures, is suggested to interact with DUA1, enhancing its levels. In Dular, a defective DUA1 protein affects the editing of rps8-182, causing the dysfunction of downstream photosynthetic proteins and inhibition of chlorophyll synthesis, and leads to leaf bleaching [14]. Overall, the chilling sensitivity of Dular is closely related to functional defects in DUA1. However, to date, no study has examined the *Lsi1*-overexpression-mediated loss of hypothermic sensitivity in Dular seedlings. Additionally, the relationship between *Lsi1* and DUA1, and the underlying mechanism that leads to the reversal of chilling sensitivity in Dular plants are largely unknown. Accordingly, in this study, we used proteomics and examined physiological responses to decipher the mechanism of *Lsi1*-overexpression-mediated chilling tolerance in rice. Additionally, we employed DNA-pulldown, co-immunoprecipitation (Co-IP), and bimolecular fluorescent complementary (BiFC) assays to determine the connection between *Lsi1* and DUA1.

## 2. Results

### 2.1. Validation of Lsi1-Overexpressing Transgenic Dular

**Figure 1:**
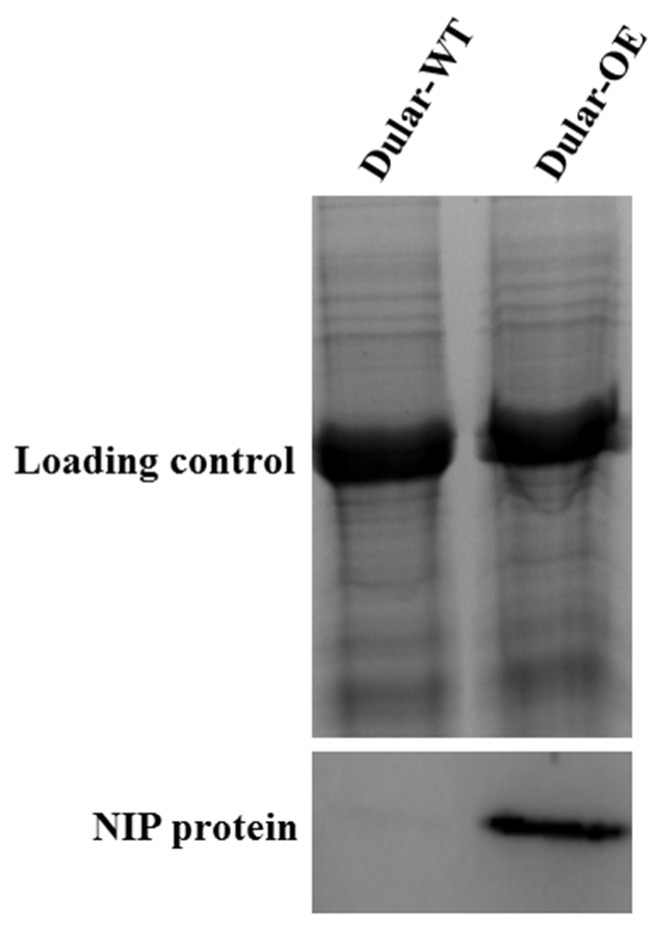
The NIP protein expression in Dular-WT and Dular-OE leaves detected by Western blot.

We detected the *Lsi1*-gene-encoded NIP protein in the leaves of Dular-OE, but not in the Dular-WT (Figure 1). Through Western blotting, the expression of the *Lsi1* gene and NIP protein in the transgenic lines was validated.

### 2.2. Cold Response of Dular-WT and Dular-OE

**Figure 2:**
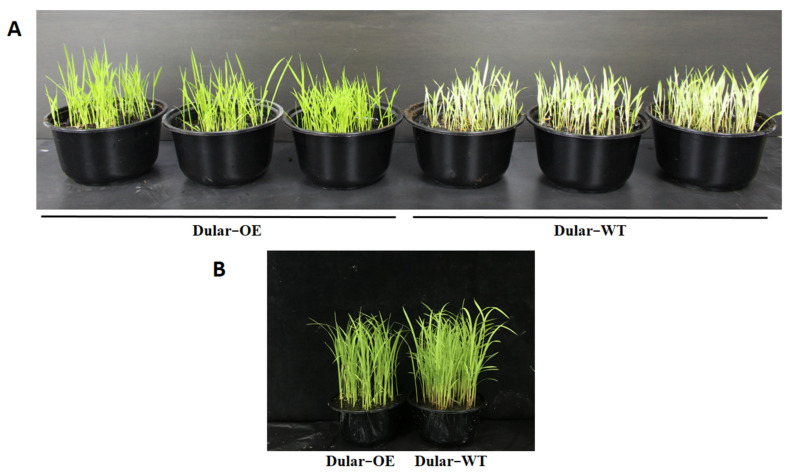
(**A**) Responses of Dular-WT and Dular-OE accessions to low temperature. (**B**) Responses of Dular-WT and Dular-OE accessions to normal temperature.

**Figure 3:**
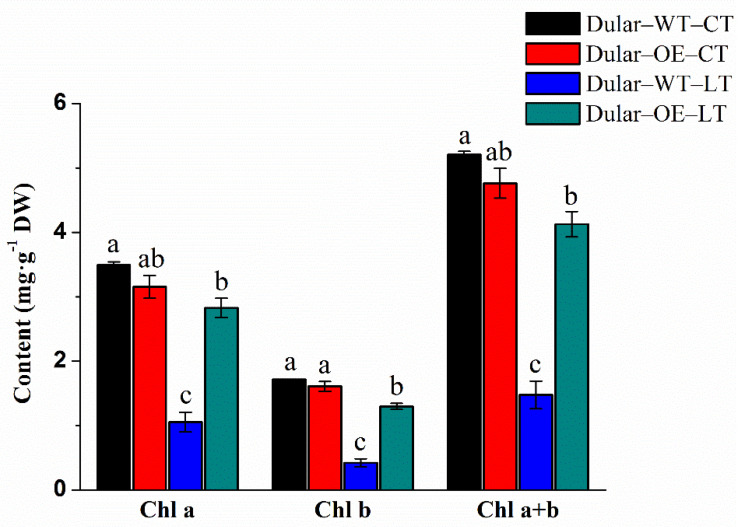
The chlorophyll content in rice leaves. Columns with different letters are significantly different (LSD test, *p* < 0.05). Error bars are the standard error (±SE) of three biological replications. DW: dry weight; CT: normal temperature; LT: low temperature.

**Figure 4:**
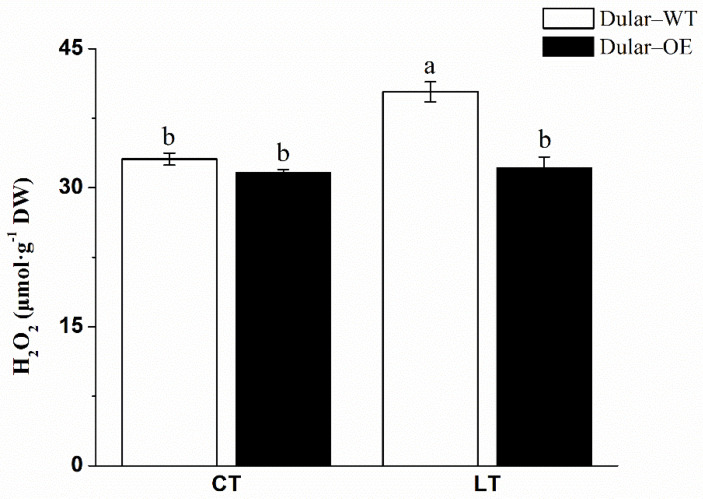
The H_2_O_2_ content in rice leaves. Columns with different letters are significantly different (LSD test, *p* < 0.05). Error bars are the standard error (±SE) of three biological replications. DW: dry weight; CT: normal temperature; LT: low temperature.

**Figure 5:**
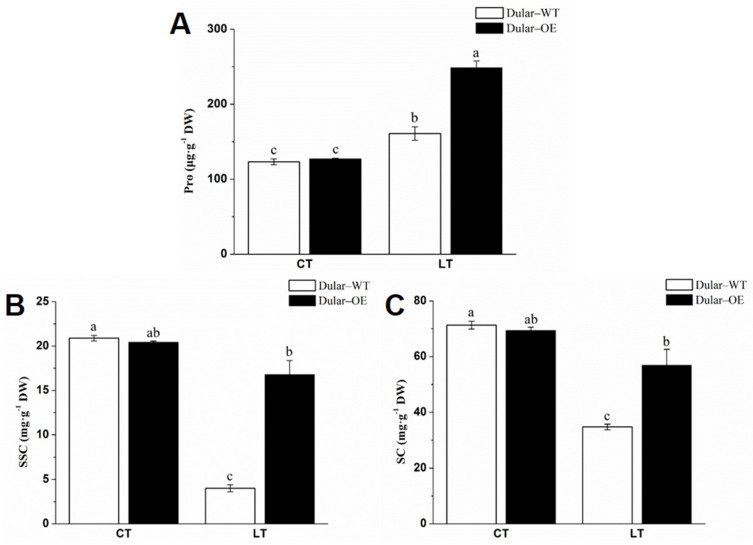
The non-structural carbohydrates and proline content in the rice leaves. (**A**) The proline content in the rice leaves. (**B**) The soluble sugar content in the leaves. (**C**) The starch content in the leaves. Columns with different letters are significantly different (LSD test, *p* < 0.05). Error bars are the standard error (±SE) of three biological replications. DW: dry weight; CT: normal temperature; LT: low temperature; SSC: soluble sugar content; SC: starch content.

Under low temperatures (15/10 °C, day/night), the leaves of Dular-OE were bright green, while Dular-WT showed pale seedlings. However, under normal temperatures, both were bright green plants (Figure 2), indicating an effect of low-temperature stress only in Dular-WT. Furthermore, under low-temperature stress, the content of chlorophyll a, b, and a + b were significantly higher (168.06, 208.53, and 179.58%, respectively) in Dular-OE than in Dular-WT (Figure 3). Additionally, the hydrogen peroxide (H_2_O_2_) content was significantly higher in Dular-WT (40.36 μmol·g^−1^·DW) than in Dular-OE (32.17 μmol·g^−1^·FW), with an increase of 25.46% (Figure 4), while the contents of superoxide dismutase (SOD), peroxidase (POD), and catalase (CAT) were significantly higher in Dular-OE than in Dular-WT, with increases of 49.80, 84.74, and 82.27%, respectively (Figure S1). Moreover, in Dular-OE, the contents of proline (Pro) and non-structured carbohydrates (NSC), including the soluble sugar content (SSC) and starch content (SC)) were significantly higher than those in Dular-WT, showing increases of 54.51, 319.04, and 63.20%, respectively (Figure 5). These results indicate that the Dular-OE seedlings could have a stable cell-membrane function. Therefore, this structural change could be the reason for enhancing levels of antioxidant enzymes, increasing Pro and NSC contents, and improving resistance of Dular-OE seedlings to low temperature, compared to that in Dular-WT.

### 2.3. Differential Protein Expression in Dular-WT and Dular-OE in Response to Low-Temperature Stress

**Figure 6:**
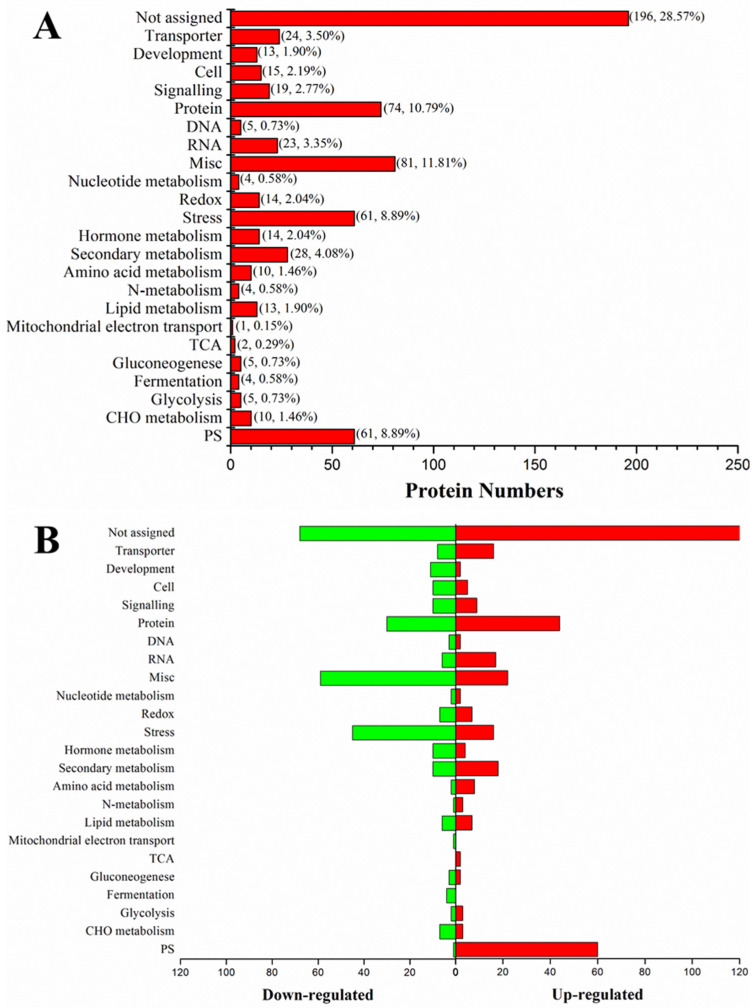
The functional pathway analysis of differential expression proteins in Dular-WT and Dular-OE. (**A**) Proteins function divided into 24 categories. (**B**) Sidewise bar graph depicting up-and down-regulating proteins.

Next, we used quantitative proteomics to analyze the protein changes in leaves in response to low-temperature stress. Raw data for this study can found in the Supplementary Materials. The PCA result depicted the high reproducibility in proteomics data (Figure S2). A total of 7070 proteins were identified in rice seedling leaves, including 676 differentially expressed proteins; 376 were upregulated and 300 were downregulated (Figure S3). The functional analysis of these differential proteins with the MapMan software revealed a total of 686 annotations, indicating their roles in multiple pathways. Accordingly, these proteins could be divided into 24 categories (Figure 6A, Table S1): PS (61, 8.89%), CHO metabolism (10, 1.46%), glycolysis (5, 0.73%), fermentation (4, 0.58%), gluconeogenesis (5, 0.73%), TCA (2, 0.29%), mitochondrial electron transport (1, 0.15%), lipid metabolism (13, 1.90%), N-metabolism (4, 0.58%), amino acid metabolism (10, 1.46%), secondary metabolism (28, 4.08%), hormone metabolism (14, 2.04%), stress (61, 8.89%), redox (14, 2.04%), nucleotide metabolism (4, 0.58%), misc. (81, 11.81%), RNA (23, 3.35%), DNA (5, 0.73%), protein (74, 10.79%), signaling (19, 2.77%), cell (15, 2.19%), development (13, 1.90%), transporter (24, 3.50%), and not assigned (196, 28.57%). The further comparison of the protein responses between the two rice lines revealed that the several signaling pathways, photosynthesis, energy metabolism, and protein synthesis were significantly upregulated in Dular-OE compared to Dular-WT (Figure 6B).

Moreover, 18 proteins were involved in various signaling pathways, including receptor kinases, calcium signaling, G proteins, and light signaling. Additionally, four carboxy-lyase proteins (carboxy-lyase; calcium-binding protein, CAST; guanosine-3, 5-bis 3-pyrophosphohydrolase; and calcium-binding protein, CML10) involved in the calcium-signaling system were upregulated. Likewise, RPT2-like protein and TIC62 proteins involved in the optical signaling pathway were significantly upregulated. These results indicated that Dular-OE better senses a low temperature and transmits it through calcium and light-signaling pathways to achieve chilling tolerance. Here, we found 61 differentially expressed proteins (DEPs) involved in the photosynthetic pathway (Figure S4), including photoreaction (45 upregulated), photorespiration (4 upregulated), and Calvin cycle (11 upregulated and 1 downregulated). A total of 24 proteins (chlorophyll a-b binding protein, photosystem II polypeptide, oxygen-evolving enhancer protein, etc.) are involved in photosystem II in the reaction to light, and 8 proteins (including photosystem I reaction center subunit) participate in photosystem I. A total of 13 proteins (including the cytochrome b6-f complex iron-sulfur subunit and ATP-synthase epsilon chain) participate in the function of cytochrome b6/f, ATP synthase, and electron carriers in the photoperiod reaction stage. 4-nitrophenylphosphatase, hydroxyacid oxidase, hydroxy-pyruvate reductase, and protein kinase C630.09c that were involved in photorespiration were upregulated. Ribulose bisphosphate carboxylase, phosphoglycerate kinase, glyceraldehyde-3-phosphate dehydrogenase, fructose-bisphosphate aldolase, and phosphoribulokinase participated in the Calvin cycle. Overall, the above results indicate that Dular-OE is well capable of sensing and responding to low-temperature signals, especially via upregulating photosynthesis, compared to Dular-WT. Notably, the NSC and chlorophyll contents also validated this conclusion.

Under low-temperature stress, the primary metabolism of Dular-OE was significantly upregulated compared to that of Dular-WT, producing more energy from amino acid and protein metabolism. Twelve DEPs were involved in energy metabolism, including the TCA cycle, glycolytic pathway, and gluconeogenesis. Two proteins were involved in the TCA cycle, while five proteins were involved in the glycolytic pathway. All these proteins were upregulated, while the other five DEPs involved in gluconeogenesis were downregulated. Additionally, ten DEPs involved in amino acid metabolism, including eight upregulated and two downregulated, were identified in Dular-OE. A total of 74 DEPs were involved in plant protein metabolism, including 44 upregulated and 30 dow-regulated proteins, involved in aspects such as ribosome synthesis, chloroplast synthesis, mitochondrial synthesis, and protein folding. A total of 31 proteins (21 upregulated, 10 downregulated) were involved in ribosome synthesis. One upregulated protein was involved in the mitochondrial synthesis, and two upregulated proteins were involved in chloroplast synthesis. These results indicate that *Lsi1* overexpression enhanced chloroplast, mitochondrion, and ribosome synthesis in Dular-OE, which effectively alleviated the potential damage caused by low-temperature stress.

Additionally, compared with Dular-WT, the secondary metabolism in Dular-OE was significantly enhanced, with regards to isoprene (terpenes), lignin, and flavonoid synthesis (Figure S5). The enhancement of secondary metabolic pathways certainly aids in the hypothermic resistance of Dular-OE. The activity of antioxidant enzymes was also significantly higher in Dular-OE.

### 2.4. Effects of Silicon on Rice Growth

Next, we examined the effect of silicon on chilling tolerance. We found that the Dular-WT plants showed a pale phenotype, while the Dular-OE plants exhibiting strong resistance to low-temperature stress were bright green at normal, half, or zero concentrations (without) of silicon (Figure S6). Furthermore, at 0, 0.35, and 0.70 mM Si concentrations, the chlorophyll a, b, and a + b content in Dular-OE was significantly higher than in Dular-WT rice, showing an increase of 81.92, 84.33, and 120.14%, respectively (Figure S7). Additionally, at the three different silicon concentrations, the Si content in the rice leaves/roots increased with an increase in the silicon concentration, in both Dular-WT and Dular-OE; however, the Si content was significantly higher in Dular-OE than in Dular-WT under the same treatment conditions (Figure S8). We found that *Lsi1* overexpression enhanced the absorption of silicon in Dular-OE. However, at a 0.70 mM silicon concentration, the leaf Si content was significantly higher in Dular-WT than in Dular-OE at 0 mM and 0.35 mM silicon concentrations. Furthermore, Dular-WT showed pale seedling at 0.70 mM Si, while Dular-OE was bright green even at 0 and 0.35 mM Si. This shows that although the overexpression of *Lsi1* increased silicon absorption, it was not the prime reason for the chilling resistance.

### 2.5. Subcellular Localization of NIP Protein

The p2300-*Lsi1*-eYFP recombinant plasmid was transfected into tobacco mesophyll cells, cultured at 26 °C for 48 h. To examine the subcellular localization of the *Lsi1*-gene-encoded protein NIP, laser scanning confocal microscopy was used to compare the data with those for the no-load GFP fluorescent sample (control). Fascinatingly, we found that the green fluorescence was evident in the cell membrane (Figure S9), indicating that NIP is localized in the cell membrane.

### 2.6. Identification of Transcription Factors Binding to the Lsi1 Promoter

The total proteins extracted from Dular-WT and Dular-OE leaves were incubated with biotin-labeled promoters and the α and β subunits of ATP synthase. After mild centrifugation, the proteins bound to promoter regions were analyzed by SDS-PAGE. The results were compared to those for the controlled samples, which were incubated with biotin-unlabeled promoters and proteins. The LC-MS/MS analysis of the DNA-pulldown samples (Figure S10) revealed interactions of the *Lsi1* promoter with six transcription factors, including high mobility group protein (HMG1), PUR ALPHA-1 (PURA1), histone-lysine N-methyltransferase (SUVH1), zinc finger C-x8-C-x5-C-x3-H type family protein (ZF-C3H), zinc knuckle family protein (ZFP), and WRKY4 (Table S2) in both Dular samples.

### 2.7. Validation of Transcription Factors Binding to the Lsi1 Promoter

**Figure 7:**
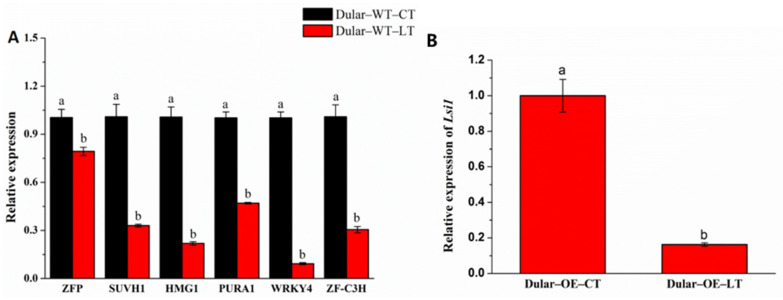
(**A**) The qPCR result of the transcription factor in Dular-WT low and normal temperature. (**B**) The qPCR result of the *Lsi1* gene in Dular-OE low and normal temperature. Columns with different letters are significantly different (LSD test, *p* < 0.05). Error bars are standard error (±SE) of three biological replications. CT: normal temperature; LT: low temperature.

Next, we used qPCR to validate the expression levels of six transcription factors that bound to the *Lsi1* gene promoter (Figure 7A). The expression of ZFP, SUVH1, HMG1, PURA1, WRKY4, and ZF-C3H was downregulated in Dular-WT under the low temperature as compared to that in Dular-WT under normal temperature. The expression of the *Lsi1* gene also downregulated in Dular-OE under the low temperature compared to that in Dular-OE under normal temperature (Figure 7B). These results indicate that under low-temperature stress, ZFP, SUVH1, HMG1, PURA1, WRKY4, and ZF-C3H could positively regulate the expression of the *Lsi1* gene.

### 2.8. Bioinformatics Screening of Transcription Factors

We used the PROMO and JASPAR databases to screen transcription factors for the *Lsi1* promoter (Tables S3 and S4). Meanwhile, based on the DNA-pulldown results (Table S5), we identified the key transcription factor, HMG1. qPCR revealed that HMG1 could enhance the expression of *Lsi1* under low-temperature stress and regulated the related downstream pathways.

### 2.9. Mass-Spectrometry Identification of NIP Interacting Partners

**Figure 8:**
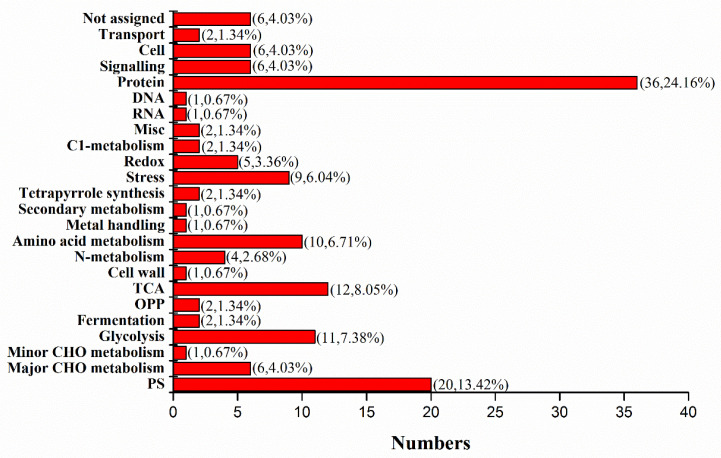
The functional classification of NIP client proteins.

Using LC-MS/MS and protein database retrieval, a total of 142 potential interacting partners of NIP were identified in Dular-OE (Figure S11). The MapMan data analysis software was used to classify the functions of these interacting proteins, and 149 protein functional annotations were obtained. The interacting partners were mainly involved in the following pathways (Table S6): PS (20, 13.42%), major CHO metabolism (6, 4.03%), minor CHO metabolism (1, 0.67%), glycolysis (11, 7.38%), fermentation (2, 1.34%), OPP (2, 1.34%), TCA (12, 8.05%), cell wall (1, 0.67%), N-metabolism (4, 2.68%), amino acid metabolism (10, 6.71%), metal handing (1, 0.67%), secondary metabolism (1, 0.67%), tetrapyrrole synthesis (2, 1.34%), stress (9, 6.04%), redox (5, 3.36%), C1-metabolism (2, 1.34%), misc. (2, 1.34%), RNA (1, 0.67%), DNA (1, 0.67%), protein (36, 24.16%), signaling (6, 4.03%), cell (6, 4.03%), transport (2, 1.34%), and not assigned (6, 4.03%) (Figure 8). These are directly or indirectly regulated by NIP, suggesting that the overexpression of *Lsi1* in the transgenic rice leaves influences signaling pathways, photosynthesis, energy metabolism, the stress response, and protein synthesis. Importantly, these are also consistent with the findings of quantitative proteomics.

### 2.10. The Interaction Patterns of NIP Proteins

*Lsi1*-YFP-N and ATP-α-YFP-C, as well as *Lsi1*-YFP-N and ATP-β-YFP-C co-transfections in agrobacterium-infected tobacco mesophyll cells resulted in yellow fluorescence. However, *Lsi1*-YFP-N and YFP-C, ATP-α-YFP-C and YFP-N, ATP-β-YFP-C and YFP-N, and YFP-N and YFP-C failed to do so (Figure S12). These results indicated that the *Lsi1* interacted with the α and β subunits of ATP synthase. Furthermore, qPCR revealed that the α and β subunits of ATP synthase were significantly upregulated in Dular-OE compared to in Dular-WT, showing increases of 396.51 and 81.75%, respectively (Figure S13). It seems that the NIP protein’s interaction with ATP synthase and regulation of downstream proteins may compensate for the abnormal function of the *DUA1* gene in the Dular plants, which is the key reason for the loss of chilling sensitivity in the Dular-OE plants.

**Figure 9:**
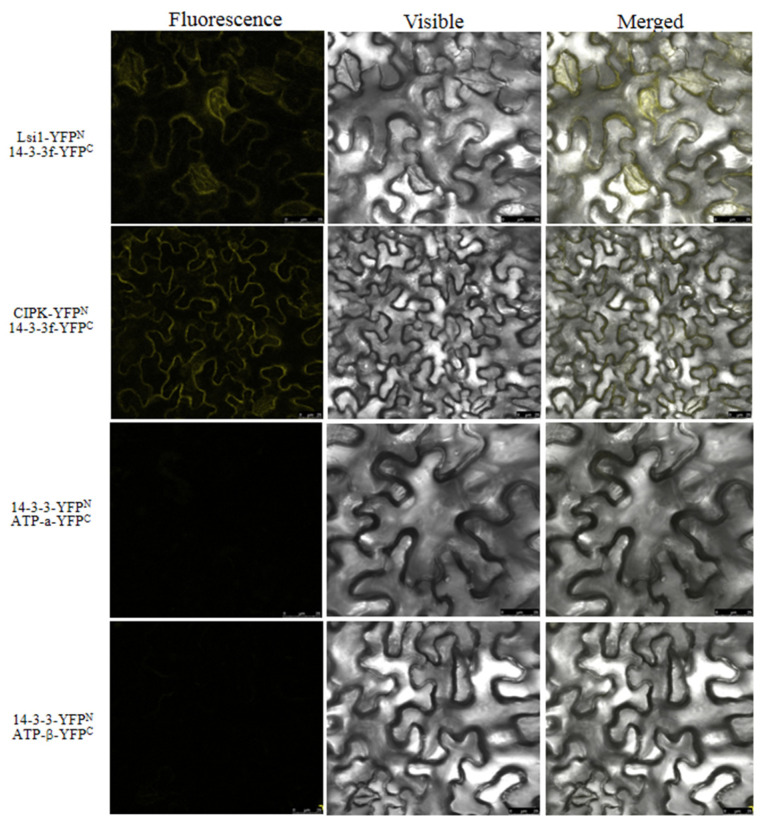
The BiFC confirmed the interaction proteins between 14-3-3f and NIP, 14-3-3f and CIPK.

**Figure 10:**
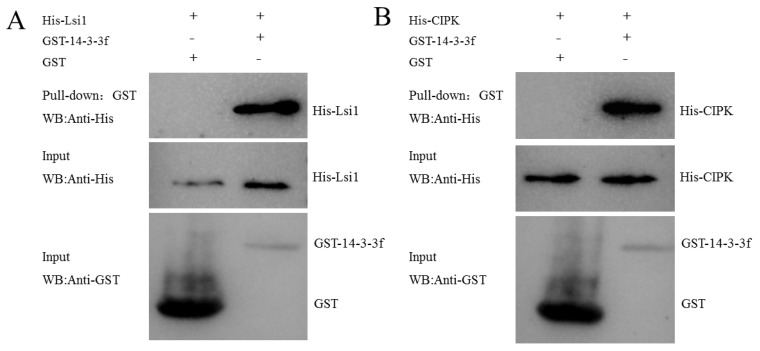
Confirmed protein interaction proteins via Western blot. (**A**) The interaction between the *Lsi1* and 14-3-3f. (**B**) The interaction between the 14-3-3f and CIPK.

**Figure 11:**
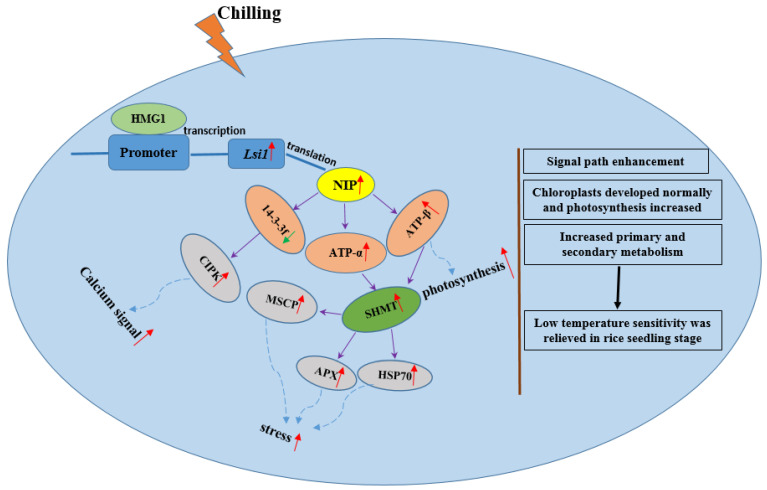
Molecular mechanism of overexpressing *Lsi1* in rice accession Dular for relieving the strong sensitivity to low temperature. Purple arrow indicates the interaction between proteins. Red arrow shows upregulation. Green arrow depicts downregulation. Dotted arrow represents protein effect on the pathway.

Moreover, we found that yellow fluorescence was evident in both the cell membrane and ER upon transfection with *Lsi1*-YFP-N and 14-3-3f-YFP-C, indicating that NIP interacted with 14-3-3f. However, no yellow fluorescence was detected upon transfection with 14-3-3f-YFP-N and ATP-α-YFP-C, or 14-3-3f-YFP-N and ATP-β-YFP-C (Figure 9), suggesting that there was no direct interaction between 14-3-3f and the α and β subunits of ATP synthase. We also identified the interaction between 14-3-3f and CBL interacting protein kinase (CIPK protein) (Figure 9), which was also confirmed by GST-pulldown (Figure 10). Western blotting showed that, under low-temperature stress, 14-3-3f was downregulated, while CIPK was upregulated in Dular-OE compared with Dular-WT (Figure S14). These results suggest that NIP indirectly enhanced the CIPK protein expression by negatively regulating the 14-3-3f protein’s function, thereby inducing the calcium-signaling pathway. Overall, these results indicated that the overexpression of *Lsi1* enhances the interaction between NIP and downstream proteins to effectively overcome the physiological barrier caused by the functional loss of the *DUA1* gene (Figure 11), which is also in agreement with the results of the physiological tests (antioxidant enzymes, pro contents, and NSC).

## 3. Discussion

### 3.1. Overexpression of Lsi1 Enhanced Chilling Tolerance in Dular-OE

The cold adaptation process plays a significant role in the survival of chilling-tolerant plants exposed to low-temperature stress. Low-temperature stress increases ROS, which damage proteins, carbohydrates, and DNA, disrupting cell functions and leading to cell senescence and then death [15]. The antioxidant system in plants controls ROS levels [16]. Here, we observed that the H_2_O_2_ content in Dular-OE was significantly lower than those in Dular-WT, while the contents of SOD, POD, and CAT were significantly higher than in Dular-WT, indicating that the Dular-OE could effectively eliminate ROS and alleviate low-temperature-induced injury. Under low-temperature stress, higher plants maintain the cell osmotic pressure by increasing the contents of proline and soluble sugars. Proline biosynthesis is activated, while its degradation is inhibited, leading to proline accumulation in cells. In favorable conditions, proline is quickly decomposed and utilized [17]. Likewise, soluble sugars can increase the amount of cell fluid, reducing the freezing point of the cytoplasm, countering excessive dehydration and maintaining the biological activity of proteins. We showed that the chilling tolerance in Dular-OE plants was significantly better than that in Dular-WT. Quantitative proteomics revealed that the signaling pathways’ photosynthesis, energy metabolism, and protein synthesis were significantly enhanced in Dular-OE plants under low-temperature stress. A comparative study on transcriptome showed that the differentially expressed genes (DEGs) between Dular-WT and Dular-OE were significantly enriched in the biosynthesis of secondary metabolites, when the two rice lines were grown under natural condition; whereas the DEGs were mainly involved in the photosynthesis, RNA transport, and proteasome, under the chilling stress. The leaf phenotype between Dular-WT and Dular-OE was also obviously different when the two rice lines were exposed to chilling stress, which indicated that Dular-OE was more tolerant to the chilling [2]. Our studies here further indicated the regulation network of NIP, which was found by the comparative analysis of the DEPs between Dular-WT and Dular-OE.

Light plays an active role in plant growth and stress tolerance [18,19]. Studies showed that RTP2 (root phototropism 2) modulated plant phototropism through NPH3 (non-phototropic hypocotyl 3) [20]. Also, Tic62 forms a photosynthetic regulator with the ferredoxin-NADP (H) redox enzyme and plays a role in chloroplast redox balance and energy metabolism [21,22,23]. In the present proteomic studies, we found that both RPT2 and Tic62 were upregulated in Dular-OE, which could be the reason for the enhanced photosynthesis and light-signal-transduction pathways under low-temperature stress.

The proteins involved in primary metabolic pathways, such as ribosome synthesis, chloroplast synthesis, mitochondrial synthesis, and protein folding, were upregulated in Dular-OE. Ribosome synthesis is the key for plant growth and environmental adaptation [24]. However, many ribosomal proteins are also involved in DNA replication, transcription, translation, DNA repair, apoptosis regulation, and other processes [25,26,27,28]. A proteomic comparison of cold-sensitive rice 9311 and cold-tolerant rice DC90 under low-temperature stress showed that the upregulation of photosynthetic and ribosomal proteins enhanced the chilling tolerance in DC90 [29]. Two important proteins associated with chloroplast synthesis that target mitochondria (IAP100 and TOC159) were upregulated in Dular-OE. IAP100 is a membrane protein involved in the intramural protein import in chloroplasts and assists in protein folding in the chloroplast cytoplasm [30]. Most chloroplast proteins are synthesized in the cytoplasm as high-molecular-weight preproteins that are introduced through the transformation between the outer chloroplast mode (TOC) and inner membrane (TIC). TOC159, a major receptor protein, directly binds to the preprotein through the polymeric GTPase domain and introduces them into the chloroplast [31]. Interestingly, these proteins are associated with ribosome synthesis, chloroplast synthesis, and mitochondrial synthesis and were found to be upregulated in Dular-OE.

Moreover, proteins involved in secondary metabolism including isoprene, lignin, flavonoid synthesis, fannigate phosphate synthetase, redox enzymes, terpene synthetase of the mevalonate pathway, carotenoids, and terpenoids in the isoprenoid synthesis pathway were found to be upregulated in Dular-OE under low-temperature stress. These pathways play an important role in plant stress tolerance [32]. The upregulation of the isoprenoid pathway in Dular-OE improves chilling tolerance. Cell-membrane damage is an important cause of the chilling injury in rice. Improved lignin synthesis in Dular-OE seedlings is beneficial for the thickening and lignification of rice cell walls, which prevents cell-membrane damage under low-temperature stress. Additionally, lignin, a complex phenolic substance, can significantly eliminate free radicals in rice. Flavonoids are important secondary metabolites that play an important role in plant stress resistance [33]. Excessive ROS create oxidative pressure that induces the accumulation of flavonoids, which protect cells from oxidative damage by quenching ROS [34].

### 3.2. Enhanced Silicon Uptake Is Not the Reason for Chilling Tolerance in Dular

Physiological and quantitative proteome analysis showed that *Lsi1* overexpression led to significant differences in chilling tolerance between the two plant strains. This was consistent with our previous reports [2,7,13]. However, these results do not sufficiently reveal the underlying molecular mechanisms and regulatory networks responsible for chilling tolerance in Dular rice. Here, we found that Dular-WT, even with a high Si content (0.70 mM) showed cold-damaged pale seedlings, while Dular-OE was resistant to low temperature. The leaves of the Dular-OE were all bright green at normal and half Si concentrations. Even in silica-free cultures, Dular-OE was cold-tolerant and its leaves remained bright green. This compelled us to speculate that chilling tolerance in Dular-OE may not be solely due to increased silicon absorption. The levels of the *Lsi1*-gene-encoded NIP protein were elevated. Additionally, it was localized in both the cell membrane and ER. Therefore, we concluded that the *Lsi1*-overexpression-dependent increase in silicon absorption may not be the primary cause of chilling tolerance in Dular-OE. Instead, the heterotopic expression of NIP could have new functions related to this phenomenon.

### 3.3. Molecular Pathways Induced by Lsi1 Overexpression Are the Key to Chilling Tolerance in Transgenic Dular

*Lsi1* overexpression may trigger corresponding pathways to achieve chilling tolerance in Dular-OE. The transcription factors specific to the *Lsi1* promoter were identified by DNA-pulldown. One such transcription factor, HMG1, plays an active role in DNA replication, transcription, and repair [35,36], and was found to upregulate *Lsi1* under low-temperature stress. The NIP-interacting partners, identified by Co-IP, were found to be involved in key signaling pathways such as photosynthesis, energy metabolism, the stress response, and protein synthesis. In particular, the ATP-synthase subunits and 14-3-3f were further validated by BiFC and GST-pulldown. The ATP-synthase subunits are involved in the photosynthesis and functional processes of rice chloroplasts [37]. Cui et al. showed that, under low temperature, in Dular-WT plants, the photosynthetic complex in leaf plastids showed a decrease in photosynthetic proteins (including the light system I (PSI) PsaA and PsaB subunits, light system II (PSII) D1 and D2 subunits, ATP-synthase β subunits and Hsp90), chlorophyll synthesis, and photosynthesis [14]. We believe that, under low-temperature stress, the interaction between NIP and ATP synthase regulates their expression promoting ATP synthesis and chloroplast development. Previously, we showed the ATP-synthase subunits’ interaction with SHMT, which interacts with Hsp70, APX, and MSCP to regulate chilling tolerance in rice [13]. Our findings suggest that NIP’s interaction with ATP synthase can indirectly regulate SHMT, and thereby, promote chilling tolerance.

Additionally, NIP’s interaction with 14-3-3f negatively regulates the expression of 1 -3f, which plays an important role in metabolic activities, signal transduction, the stress response, and cellular regulation [38]. Several studies have shown that plasma membrane CRPK1 induces the phosphorylation of 14-3-3, regulating CBF signaling and enhancing the hypothermic resistance in rice [39,40]. CBL-interacting protein kinase (CIPK), a serine/threonine-protein kinase, interacts with CBL, regulating downstream gene expression [3,41]. CBL and CIPK form a heterogeneous interaction network and actively participate in plant antiretroviral reactions. Zhang et al. showed that site mutations in *OsCIPK7* led to conformational changes in the kinase domain, increasing the protein kinase activity, which increased the chilling tolerance [42]. Previous studies showed that under low temperature, the *COLD1* gene triggered the influx of calcium, activating the key transcription factors that increase the chilling tolerance in rice [43]. Our results suggest that *Lsi1* negatively regulates 14-3-3f, which positively regulates CIPK, regulating the calcium-signaling pathway.

## 4. Materials and Methods

### 4.1. Plant Growth and Treatments

The genetically stable Dular-WT and Dular-OE rice lines were introduced and developed by Fujian Agriculture and Forestry University [2,7,13].

Rice seeds were treated with 25% sodium hypochlorite (NaClO) solution to disinfect for 15 min. Afterwards, the seeds were rinsed with distilled water and soaked for 48 h. Then, the seeds were put into an incubator for 24 h (85% relative humidity) with the temperature set to 28 °C in the dark. The seeds were transferred in a tray (upper surface diameter, 13 cm; lower surface diameter, 8 cm; height, 7.5 cm) upon germination. The material in the tray consisted of matrix soil and was put in the light (temperature: 28 °C, humidity: 85%, light: 20,000 lux and time: 14 h) and dark (temperature: 22 °C, humidity: 85% and time: 10 h). After the growth of 1.5 leaves, the planting conditions were adjusted in the light (temperature: 15 °C, light: 20,000 lux, time: 14 h and relative humidity: 85%) and then in the dark (temperature: 10 °C, time: 10 h and relative humidity: 85%). Meanwhile, normal planting (control group) was performed in the light (temperature: 28 °C, light: 20,000 lux, time: 14 h and relative humidity: 85%) and dark (temperature: 10 °C, time: 10 h and relative humidity: 85%). The samples were collected after the interval of 48 h after the treatment.

### 4.2. Protein Extraction and Western Blot Analysis

The total protein from the rice leaves was extracted using the trichloroacetic acid (TCA)/acetone method [44]. Briefly, a mortar precooled in liquid nitrogen with a small amount of quartz sand was used to grind 2 g of frozen (−80 °C) leaf samples from the Dular-WT and Dular-OE lines. The ground powder was transferred to a 10 mL centrifuge tube containing 5 mL of protein-extraction medium (10% TCA, 90% acetone, and 0.07% β-mercaptoethanol). The tube was oscillated with a vortex oscillator for 5 min and then placed in ice for 10 min (repeated thrice). After this, the sample tube was kept at −20 °C for 12 h and then centrifuged for 25 min (11,000× *g*) at 4 °C. The supernatant was discarded, and the precipitate was gently cleaned with 100% acetone (containing 0.07% β-mercaptoethanol). The centrifuge tube was transferred back to −20 °C in storage for 4 h. This step was repeated several times until the supernatant became free from pigments. Finally, the supernatant was removed. The centrifuge tube was freeze-dried, and the acetone in the precipitate was removed by rotary evaporation to finally obtain the dried protein powder. This powder was extracted using protein lysate (Thermo Fisher Scientific, Waltham, MA, USA) (1:10; 50 mM Tris, 0.1% SDS, 1% NP-40, and 150 mM NaCl, pH 7.4), and the protein concentration was determined by the Bradford method [45].

A total of 20 μg of protein was mixed with 6 μL of 5 × loading buffer, and the sample was heated for 10 min at 95 °C. Then, the samples were quickly placed on ice for 2 min and briefly centrifuged for 20 s. The proteins were resolved by SDS-PAGE and then semi-dry transferred to a membrane at 1.5 A and 75 V for 15 min. After the transfer, the membrane was cleaned with PBST for 5 min at normal temperature and then blocked in 5% skimmed milk (dissolved in PBS) for 1 h. Next, it was incubated with an appropriate amount of primary antibodies (Abmart, Shanghai, China) for 3 h at RT and then washed with PBST for 5 min, 5 times. Next, incubation was performed with secondary antibodies (Abmart, Sanghai, China) at normal temperature for 1 h. After washing, the bands were illuminated by a chemiluminescence (ECL) reaction and the images were recorded using the ChemiDoc MP Imaging System (Bio-RAD, Hercules, CA, USA).

### 4.3. Determination of Chlorophyll, H_2_O_2_, Enzyme Activity, and Pro and NSC Contents

Fresh leaves (0.2 g), treated for 48 h at low and normal temperature, from the respective rice lines, were placed in a mortar for grinding with 96% ethanol, and the volume was made up to 25 mL (three biological repeats). The absorbance of the extracted liquid at 665 and 649 nm was determined, and the concentration was calculated [7].

The fresh rice leaves (three biological repeats), treated at low and normal temperature for 48 h were ground in liquid nitrogen and suspended in 5 mL of precooled PBS (50 mM, pH 7.0). The supernatant was centrifuged at 11,000× *g* at 4 °C for 20 min, and the activity of SOD, POD, and CAT in the supernatant was determined following the previously described method [46].

Dry rice leaf samples (three biological repeats) treated at low and normal temperature for 48 h were used to determine the soluble sugar and starch contents as described by Yoshida [47].

Fresh rice leaf samples (three biological repeats) from low and normal temperature treatments, each weighing 0.5 g, were used for estimating the proline contents, as described by Khare et al., [48]. The sample absorbance at 520 nm was measured, and the concentration was calculated based on the colorimetric reaction between proline and acidic ninhydrin.

A total of 1 g of fresh plant leaves (three biological repeats) treated at a low and normal temperature for 48 h were added to 2 mL of precooled acetone to prepare the homogenate. This was centrifuged at 3000× *g* for 10 min, and the supernatant was used for estimating the H_2_O_2_ content by measuring the absorbance at 415 nm as described by Li et al., [44]. H_2_O_2_ was determined by external calibration. The standard curves of H_2_O_2_ content were developed by determining the absorbance of different concentrations of H_2_O_2_. The H_2_O_2_ content in the sample was obtained by a standard curve and calculation formula. H_2_O_2_ content in plant tissues = C ∗ V_t/_(W ∗ V_1_), C: the content of H_2_O_2_ in the extract of the sample was determined on the standard curve; V_t_: the total volume in sample extract; V_1_: the liquid volume of sample extraction was used for determination; W: fresh weight.

We first measured the enzyme activities, chlorophyll content, proline, and H_2_O_2_ in fresh weight. As low temperature affects the fresh weight, we therefore measured the water content of the samples and subtracted it from the fresh weight and found the results of enzyme activity, chlorophyll content, proline, and H_2_O_2_ in dry weight.

### 4.4. Quantitative Proteomics

The protein was extracted from Dular-WT and Dular-OE leaves that were treated at low temperatures for 48 h. Each repeated sample was considered a separate biological replicate. Each biological sample was individually incubated in lysis buffer (2 M thiourea, 7 M urea, 4% SDS, and 40 mM Tris-HCl, pH 8.5) containing 1 mM PMSF and 2 mM EDTA (final concentration) for 5 min, and then, 10 mM DTT (final concentration) was added. After vortex mixing for 15 min, the sample mixture was centrifuged at 4 °C and 13,000× *g* for 20 min. Then, 5 times the volume of precooled acetone was added with proper mixing and the mixture was kept at −20 °C for 12 h. After centrifugation at 13,000× *g* and 4 °C for 20 min, the obtained protein particles were freeze-dried and then treated with 8 M urea (in 100 mM TEAB, pH 8.0). Next, 10 mM DTT (final concentration) was added to the protein samples for 30 min at 56 °C, and 50 mM (final concentration) iodoacetamide (IAM) was added for 30 min in the dark. The samples were diluted 5 times with 100 mM TEAB, and protein was extracted for trypsin digestion (1:30 W/W) at 37 °C overnight. After the trypsin digestion, the product was acidified with 0.1% formic acid (FA), and the peptides were desalted with a preactivated (in 1 mL methanol) C18 chromatography column that was balanced with 1 mL of 0.1% FA. Next, the column was washed twice with solvent A (5% acetonitrile (ACN), 0.1% FA) and eluted with 1 mL of solvent B (80% ACN, 0.1% FA). Finally, the purified peptides were lyophilized and dissolved in 20 μL of 0.5 M TEAB.

The peptide fragments in the sample were labeled with the Isobaric Tag for Relative Absolute Quantitatio (iTRAQ) kit (Thermo Fisher Scientific, Waltham, MA, USA) and then dried. The iTRAQ samples were labeled as Dular-OE-13, Dular-WT-14, Dular-OE-15, Dular-WT-16, Dular-OE-17, Dular-WT-18, and lyophilized, accordingly. The iTRAQ-labeled samples were resolved into 12 components using the Durashell C18 (5 μm, 100 Å, 4.6 × 250 mm) using a high-performance liquid chromatography (HPLC) system (Thermo Fisher Scientific, Waltham, MA, USA). Each part, dissolved in 30 μL of 2% ACN (0.1% FA), was analyzed using the TOF 5600 high-resolution mass spectrometer (SCIEX, Sparta Township, NJ, USA). The peptide samples (5 mL) were injected into a C18 absorption column (5 μm, 100 μm × 20 mm) and eluted using a 90 min gradient program at a 300 nL/min flowrate into a C18 assay column (3 μm, 75 μm × 150 mm). For Information Dependent Acquisition (IDA), a survey scan was carried out at 250 nm, and 30 product ion scans were taken at 100 ms/scan. The MS^1^ and MS^2^ spectrum were acquired in the range of 350–1500 and 100–1500 *m*/*z*, respectively. The precursor ions were excluded from the reselected 15 s.

We employed Rice Genome Annotation Project Database (RAP-DB) (http://rice.uga.edu/, accessed on 15 April 2022) and MS/MS data were search against it in order to get peptide/protein identification information. To find target proteins, a Paragon Algorithm (SCIEX) was used, and to get rid of redundant hits. For labeling quantification, more than two unique peptides were taken. For further data analysis, we used peptides with global false discovery rate (FDR) values from a fit < 1%. The leaf proteins that significantly altered between Dular-WT and Dular-OE were selected, and the standard deviation and log2 values were calculated. Protein functional annotations were obtained from the RAP-DB, and the functional proteins were classified according to the bincodes of MapMan (https://mapman.gabipd.org/, accessed on 15 April 2022) [49].

### 4.5. Nutrient Solution Experiment

Rice seedlings were grown as described above in “Research Materials”. When the rice had grown to the 1.5 leaf stage, Dular-WT and Dular-OE were transplanted to a plastic basin containing complete nutrient solution in a 2 L volume (pH 5.8), as cold-sensitive and cold-resistant controls, respectively, with ten plants per pot. The nutrient solution was treated with different silicon concentrations (0 mM: low concentration; 0.35 mM: normal concentration; and 0.70 mM: high concentration). Each treatment was three biological repeats. The culture conditions were 15 °C for 14 h with a 20,000 lux light intensity, and 10 °C in the dark for 10 h under 85% relative humidity. Meanwhile, the control planting at normal temperature was performed at 28 °C in light for 14 h with a 20,000 lux light intensity, and 22 °C in the dark for 10 h under 85% relative humidity. After 48 h of the low-temperature treatment, the leaf phenotypic changes in each group were observed. Additionally, the chlorophyll and silicon contents were determined as described by Sadia and others [7].

### 4.6. Subcellular Localization of NIP Protein

The total RNA obtained from the Dular-OE plants was reverse transcribed into cDNA. Primers corresponding to rice *Lsi1*′*s* full-length CDS (*Lsi1*-Fusion-F and *Lsi1*-Fusion-R) were used for gene amplification, and the recombinant expression vector 35S::*Lsi1*-inGFP was constructed by fusion cloning with the pBinGFP2 vector. *Agrobacterium tumefaciens* containing the 35S::*Lsi1*-in GFP plasmid was transformed into tobacco mesophyll cells by the PEG-mediated method. The empty vector containing only the green fluorescent protein gene (35S::inGFP) was separately transferred into tobacco leaf cells. The two transformed tobacco cultured at 26 °C for 48 h. The distribution of green fluorescence in the tobacco leaf cells was observed by laser confocal microscopy, and the subcellular localization of the NIP protein controlled by the *Lsi1* gene was determined. The primers are listed in Table S7.

### 4.7. DNA-Pulldown: Transcription Factors Interacting with the Lsi1 Gene Promoter

Dular-WT and Dular-OE leaves treated at low temperature for 48 h were frozen in liquid nitrogen and then quickly ground into powder. Based on the method described by Fang et al. [13], leaf natural protein was extracted using Pi-IP solution (50 mM Tris-HCl, 150 mM NaCl, 1 mM EDTA pH 8.0, 1% Triton X-100, 1 mM PMSF, 1 × EDTA-free Protease Inhibitor Cocktail, Roche, Merck). The promoter region is 2000 bp upstream of the *Lsi1* gene CDS sequence. The 5′ end of the primer, upstream primer *Lsi1*-promoter-F, was labeled with biotin, and the length of the *Lsi1* promoter was amplified with the downstream *Lsi1*-promoter-R, a reverse primer. To identify the transcription regulators interacting with the *Lsi1* promoter, DNA pull-downs were performed as described by Fang et al. [2].

### 4.8. Bioinformatics Prediction of Lsi1 Gene-Promoter Transcription-Factor-Binding Sites

The 2000 bp upstream of the *Lsi1* gene’s CDS was used as the gene promoter, and the Promo (http://alggen.lsi.upc.es/cgi-bin/promo_v3/promo/promoinit.cgi?dirDB=TF_8.3, accessed on 15 April 2022) and JASPAR (https://jaspar.genereg.net/, accessed on 15 April 2022) databases were used for bioinformatic prediction.

### 4.9. qPCR Validation of Transcription Factors

Total RNA extracted from leaves of Dular-WT and Dular-OE, treated at normal or low temperatures for 48 h, was reversed transcribed into cDNA using the TransScript One-Step gDNA Removal and cDNA Synthesis SuperMix kit (Takara bio, Kusatsu, Shiga, Japan). The qPCR was carried out using the TransStart Tip Green qPCR Supermix kit (Bio-Rad, Hercules, CA, USA) and the Eppendorf Realplex4 (Eppendorf, HAM, DE) instrument. The qRT-PCR conditions were as follows: predenaturation at 94 °C for 30 s, denaturation at 94 °C for 5 s, annealing at 55 °C for 15 s, and extension at 72 °C for 10 s (42 cycles). The relative expression of each candidate mRNA in different samples was calculated by the 2^−^^ΔΔ^^Ct^ method. The qPCR primers are listed in Table S7.

### 4.10. NIP-Interacting Proteins

NIP-interaction proteins in both the rice lines (treated at low temperature for 48 h) were identified. Briefly, 1 g of rice leaves were thoroughly ground in liquid nitrogen and the dry powder was extracted with 1 mL of extraction buffer (50 mM Tris-HCl, 0.5 mM EDTA, 0.5% NP-40, 150 mM NaCl, 4 mM MgCl_2_, 5 mM DTT, and 1 mM PMSF, pH 7.5) by thorough mixing. The mixture was placed on ice for 30 min and oscillated every 5 min. Then, the extract was centrifuged at 11,000× *g* and 4 °C for 30 min. The supernatant was filtered using a 0.22 μm membrane to obtain a crude extract of non-denatured proteins. This was treated with antibodies (200:1) overnight at 4 °C. Then, 100 μL of protein A/G agarose was blended gently for 2 h at RT, and then, ~0.5 mL of IP buffer (25 mM Tris and 150 mM NaCl, pH 7.2) was added. The mixture was centrifuged at 2500× *g* for 3 min to obtain the supernatant. This step was repeated twice. The precipitate was washed with 0.5 mL of deionized water and centrifuged at 2500× *g* for 3 min, and the supernatant was separated. Next, 50 μL of 5 × loading buffer was added to the supernatant fraction for heating at 95 °C for 5 min. After centrifugation at 2500× *g* for 3 min, the supernatant was taken and 15 and 30 μL of protein samples were resolved by SDS-PAGE and HPLC-LTQ LC-MS/MS (Thermo Fisher Scientific, Waltham, MA, USA), respectively.

We employed RAP-DB and MS/MS data were search against it in order to get peptide/protein identification information. We used the following parameters: trypsin was taken as an enzyme and the fixed modification of methyl methanethiosulfonate of cysteine residues was set. To find target proteins, a Paragon Algorithm (SCIEX) was used, and to get rid of redundant hits. For labeling quantification, more than two unique peptide were taken. For further data analysis, we used peptides with global false discovery rate (FDR) values from a fit < 1%. The functional proteins were classified according to the bincodes of MapMan [49]

### 4.11. Bimolecular Fluorescence Complementation to Validate Protein Interactions

For the bimolecular fluorescent complementary (BiFC) analysis of protein interactions, the CDSs of the corresponding target genes were used for designing the specific upstream and downstream primers. The full-length gene was amplified by PCR using the cDNA of Dular as the template. Using a linearization enzyme, an eYFP fluorescent protein containing an N-terminal p2300 and C-terminal p2300-2YN, and p2300-2YC (restriction enzyme cutting site Spe I), respectively, were merged to obtain the *E.coli* Trans1-T1 (*Lsi1*-p2300-2YN, ATP-α-p2300-2YC, ATP-β-p2300-2YC, 14-3-3f-p2300-2YC, and CIPK-p2300-2YC. Single colonies were verified by PCR and then transferred to *Agrobacterium tumefaciens* EHA105. *Agrobacterium tumefaciens* was selected and cultured in 5 mL of LB medium (containing 50 mg/L of Kanamycin (Kan^+^) and 50 mg/L of rifampicin (Rif^+^)) at 28 °C for 16 h. When it reached an OD_600_ of 1–2, the bacterial culture was transferred to fresh LB medium (1:500; 10 mM MES, 20 μM AS, 50 mg/L Kan^+^, and 50 mg/mL Rif^+^) for growth at 28 °C for 24 h. Then, an infiltration buffer (10 mM MES, 150 μM AS and 10 mM MgCl_2_) was used to re-suspend (adjusted to OD_600_ 1.5) the harvested bacteria at 6000 g for 10 min. The suspension was kept at RT for 4 h and then mixed with *Agrobacterium tumefaciens* in equal volumes. The mixture was slowly injected into well-grown tobacco plant leaves (plants were around 2 weeks old at the 4th–5th leaf stage), and the whole leaves were covered. After 48 h, the culture was continued under normal-light conditions. The infected leaves were observed under a laser confocal microscope LSM710 (Carl Zeiss AG, Oberkochen, DE) to analyze the fluorescence distribution.

### 4.12. GST Pull-Down Assay

The GST::14-3-3f, His::*Lsi1*, and His::CIPK fusion proteins were expressed. GST::14-3-3f bound to glutathione sepharose was incubated with His::CIPK at low temperature, rinsed with 1 × PBS buffer, and then eluted in a glutathione-reducing Tris-HCl solution (pH 8.0). A 20 µL volume of eluent was mixed with 10 µL of 5 × loading buffer and heated for 10 min at 95 °C. Then, it was quickly transferred to 4 °C storage for 2 min. After mild centrifugation, the samples were analyzed by SDS-PAGE electrophoresis and Western blotting using His and GST antibodies.

### 4.13. Statistical Analysis

All the data presented in the paper are the means of three biological replicates. The differences among the treatments were calculated and statistically analyzed using the variance and the least-significant-difference multiple-range test (LSD, *p* < 0.05). The Statistical package for OriginPro 8.0 (OriginLab, Northampton, MA, USA) and the Data Processing System (DPS) version 7.05 (Zhejiang University, Hangzhou, China) were used for the statistical analysis.

## 5. Conclusions

In conclusion, under low-temperature stress, the Dular-WT plant, due to the dysfunction of the *DUA1* gene, exhibits rsp8-182 editing, which results in a decline in ribosomal protein levels, ribosome biosynthesis, photosynthetic proteins, chlorophyll synthesis, and photosynthetic ability. All these lead to pale rice seedlings with stunted growth and development. Here, we showed that the *Lsi1*-encoded NIP protein, expressed on the membranes and ER of rice leaf cells, interacts with other proteins in a manner that ameliorates the functional defects of *DUA1* in Dular-WT. The overexpressed NIP interacts with ATP synthase subunits, enhancing the light-signaling pathway, photosynthesis, and the development of chloroplasts. Moreover, NIP, through the negative regulation of 14-3-3, enhances the CIPK gene’s expression, which promotes the calcium-signaling pathways; cell membrane stability, and synthesis of organelles such as chloroplasts, mitochondria, and ribosomes, which eventually increases chilling tolerance in Dular-OE.

## Data Availability

All data generated during this study are included in this published article and its supplementary information files, and the raw data used or analyzed during the current study are available from the corresponding author on reasonable request.

## Supplementary Materials

The following supporting information can be downloaded at: https://www.mdpi.com/article/10.3390/ijms23094667/s1.
