# Supplementary material for: Deciphering the Molecular Mechanisms of Chilling Tolerance in Lsi1-Overexpressing Rice
Source: Int J Mol Sci. 2022 Apr 23;23(9):4667. doi: 10.3390/ijms23094667 (PMC9103898; doi:10.3390/ijms23094667)
Supplement: Supplementary file 1 [file ijms-23-04667-s001.zip › supliment-table and figure-1.pdf]

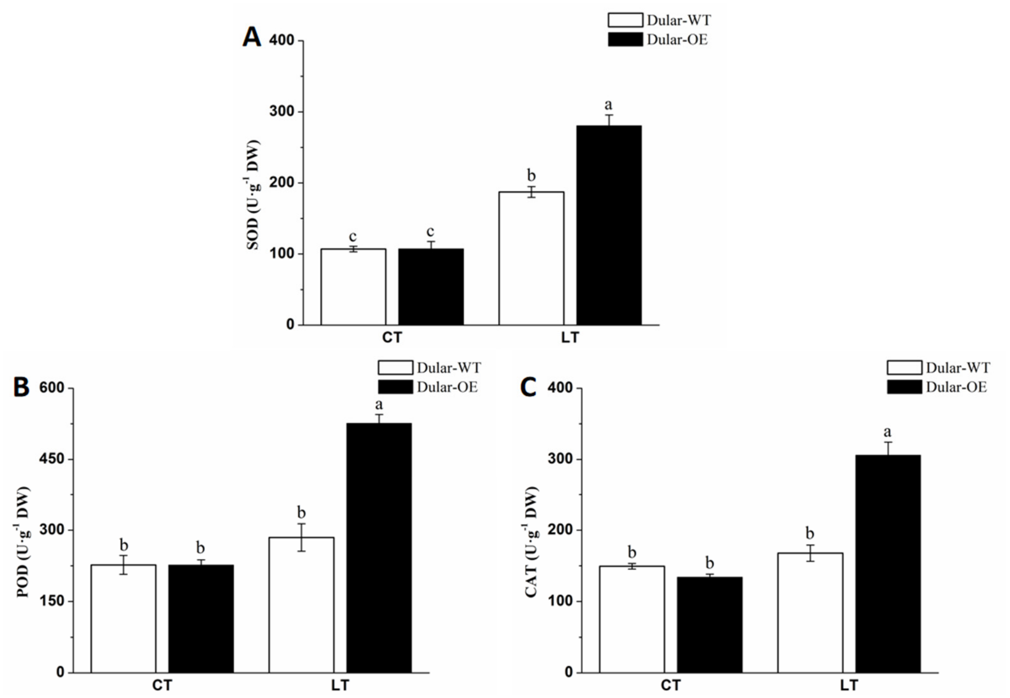

Figure S1： The protective enzyme content (SOD, POD and CAT) in rice leaves. Columns with different letters are significantly different (LSD test, $P < 0.05$). Error bars are standard error (±SE) of three biological replications. DW: dry weight. CT: normal temperature. LT: low temperature.

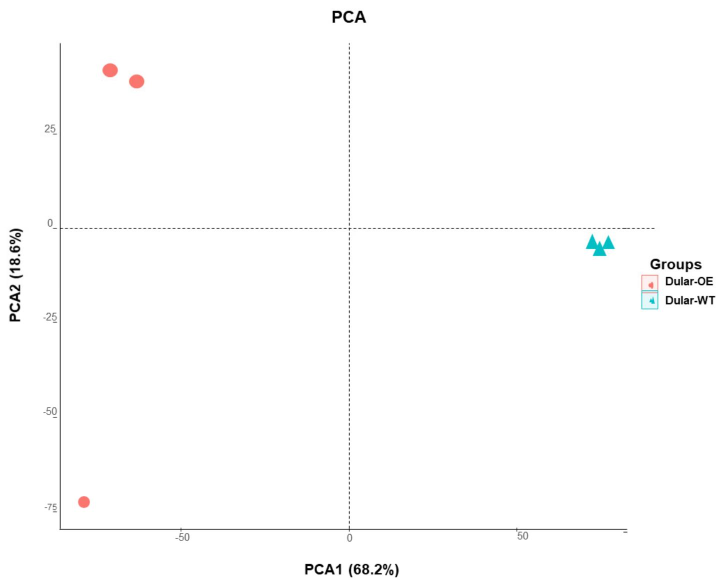

Figure S2: The PCA of rice leaf protein

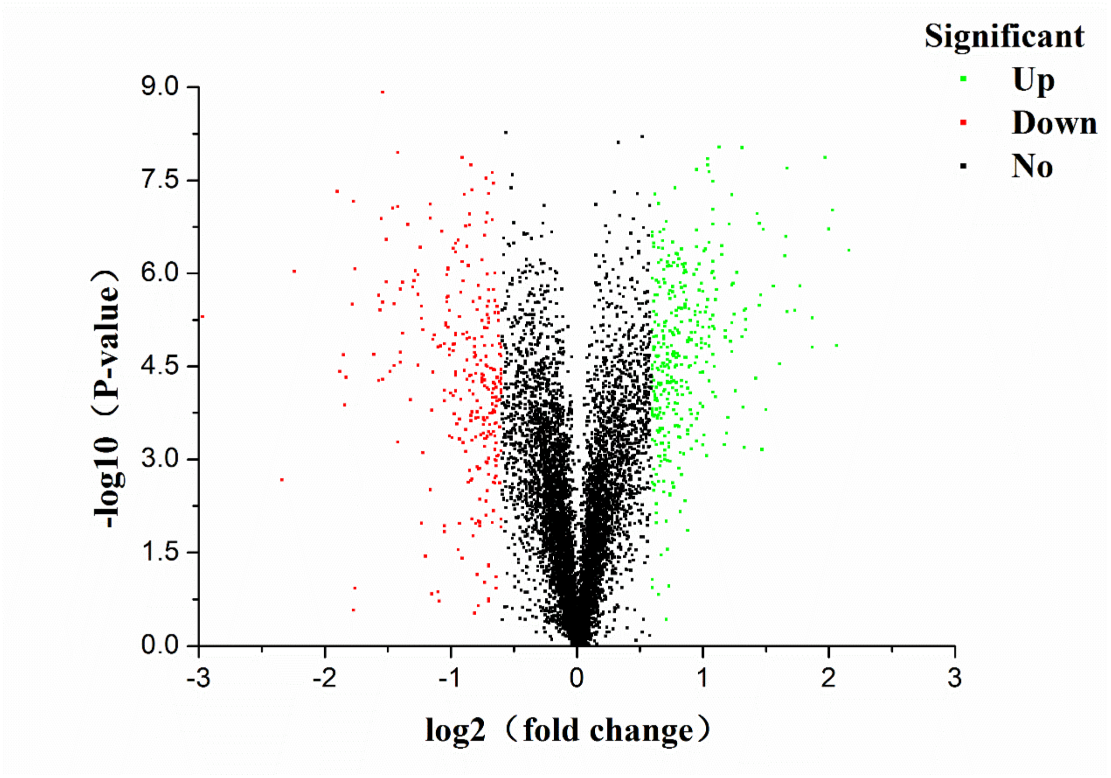

Figure S3: The volcanic map of rice leaf protein

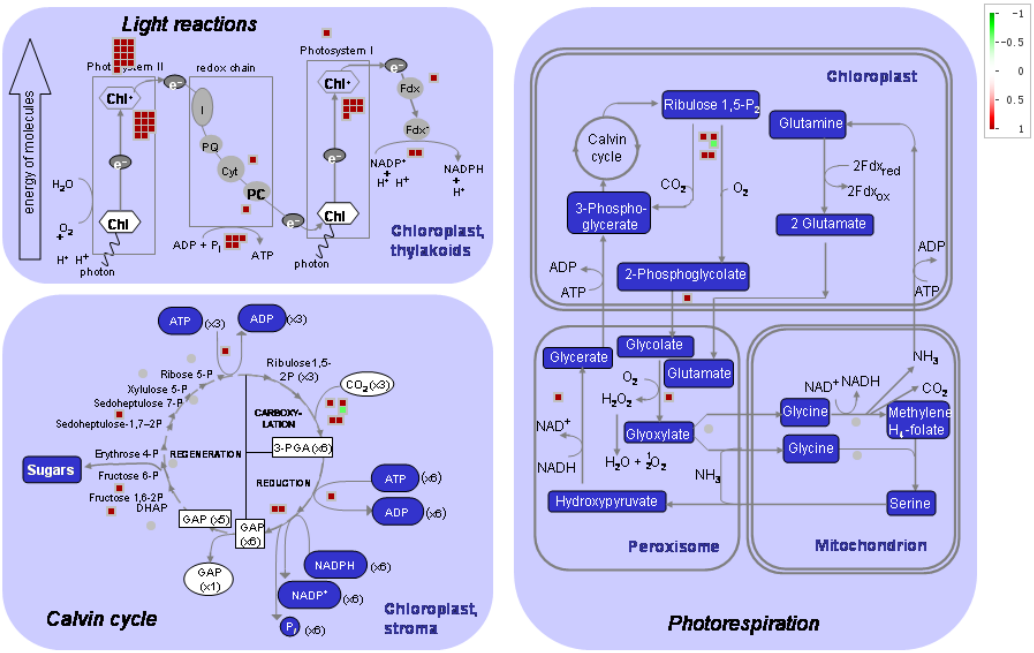

Figure S4: MapMan analysis of the protein of chlorophyll synthesis and photosynthesis under chilling

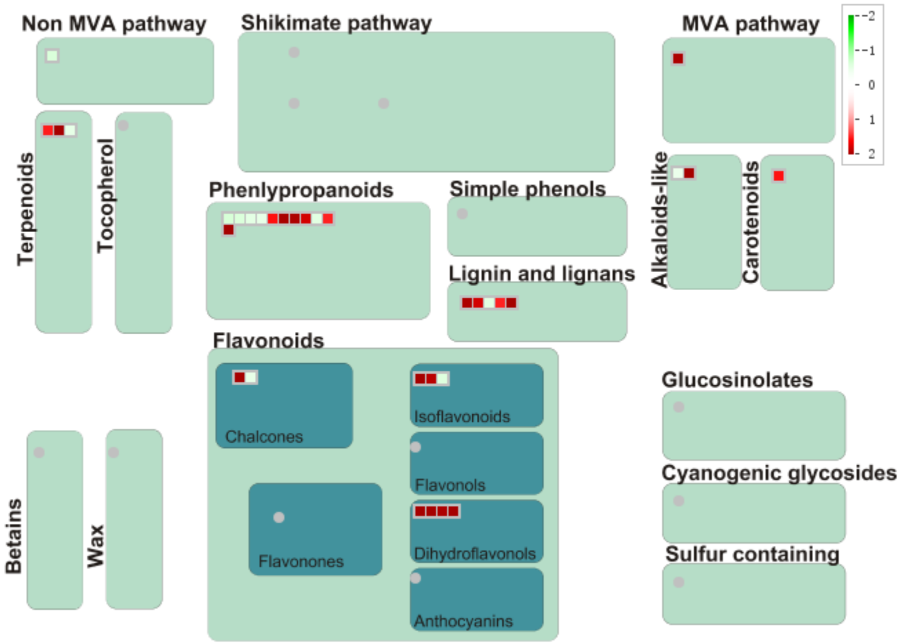

Figure S5: MapMan analysis of the protein of secondary metabolism under chilling

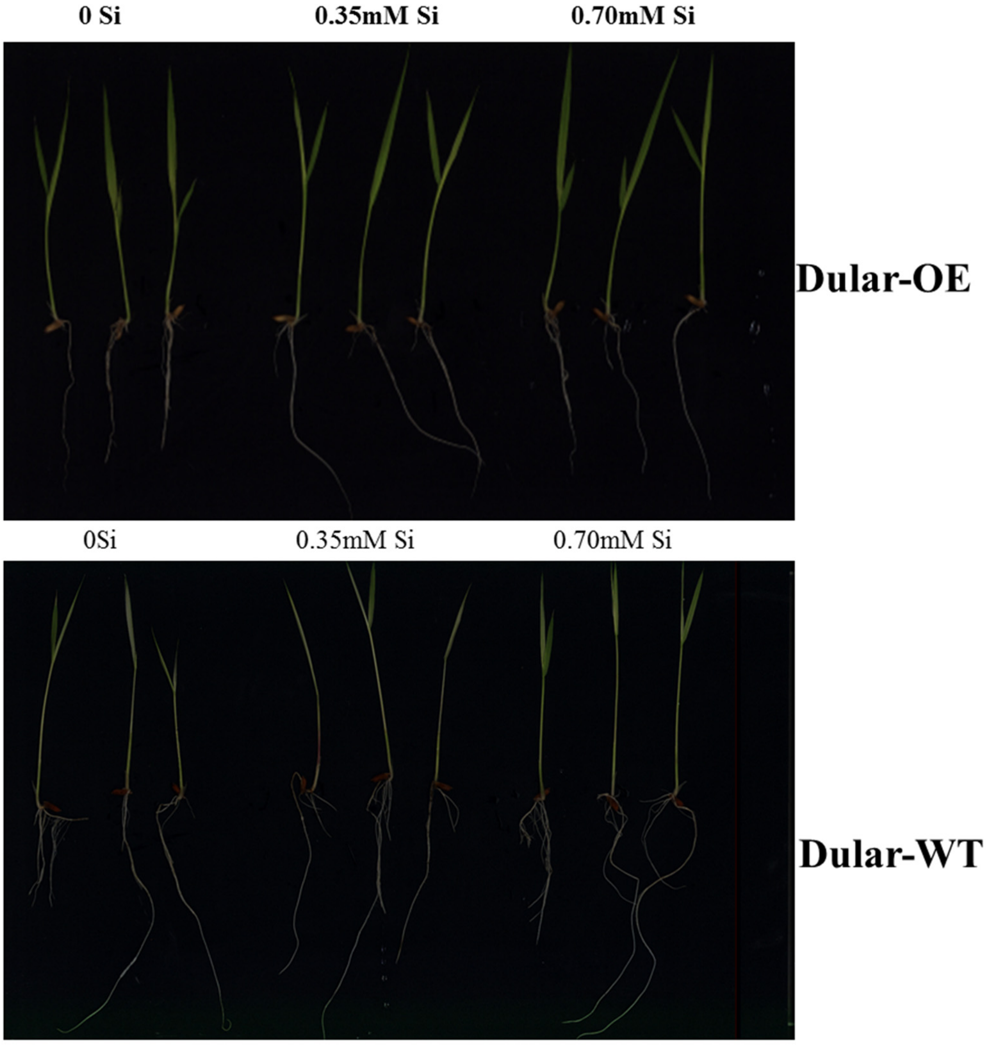

Figure S6: The leaf phenotypes of Dular-WT and Dular-OE exposed to low temperature stress in hydroponics containing different silicon nutrient concentrations

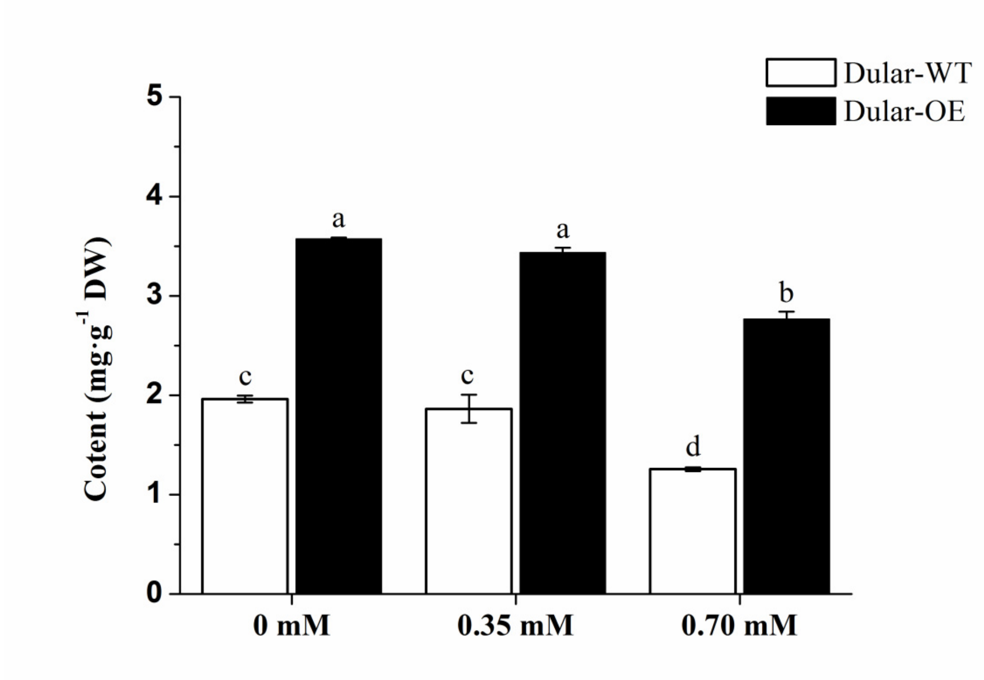

Figure S7: The leaf chlorophyll a+b contents of Dular-WT and Dular-OE exposed to low temperature stress in hydroponics containing different silicon nutrient concentrations. Columns with different letters are significantly different (LSD test, $P < 0.05$). Error bars are standard error (±SE) of three biological replications. DW: dry weight.

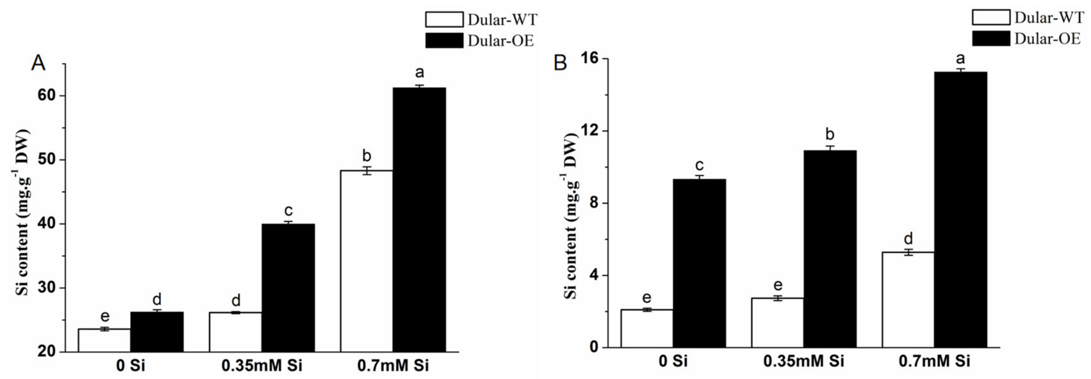

Figure S8: The determination of silicon content in Dular-WT and Dular-OE exposed to low temperature stress in hydroponics containing different silicon nutrient concentrations. Columns with different letters are significantly different (LSD test, $P < 0.05$). Error bars are standard error (±SE) of three biological replications. DW: dry weight. A: Leaf； B: Root

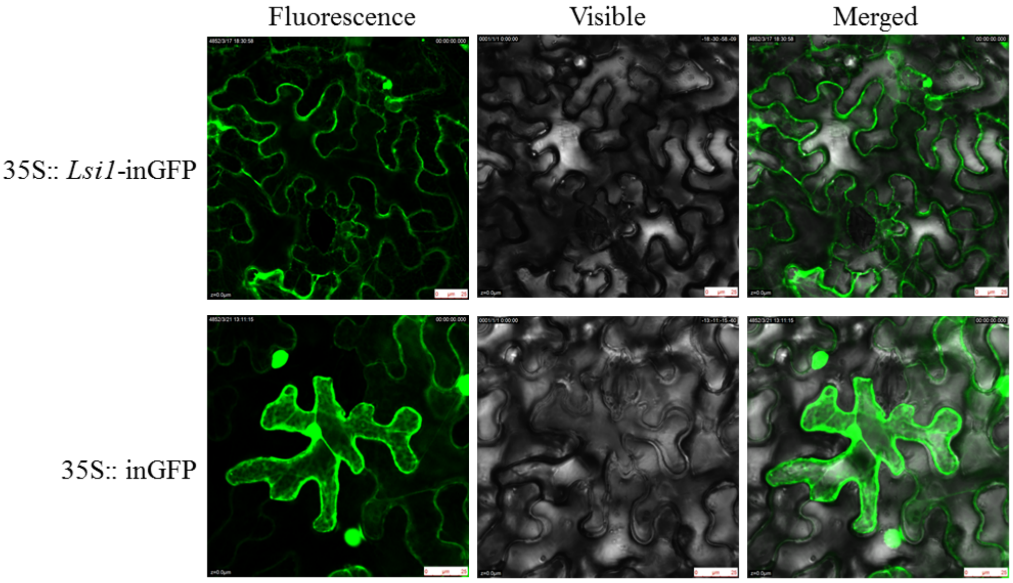

Figure S9: The NIP protein localization in sub organelles in tobacco cells

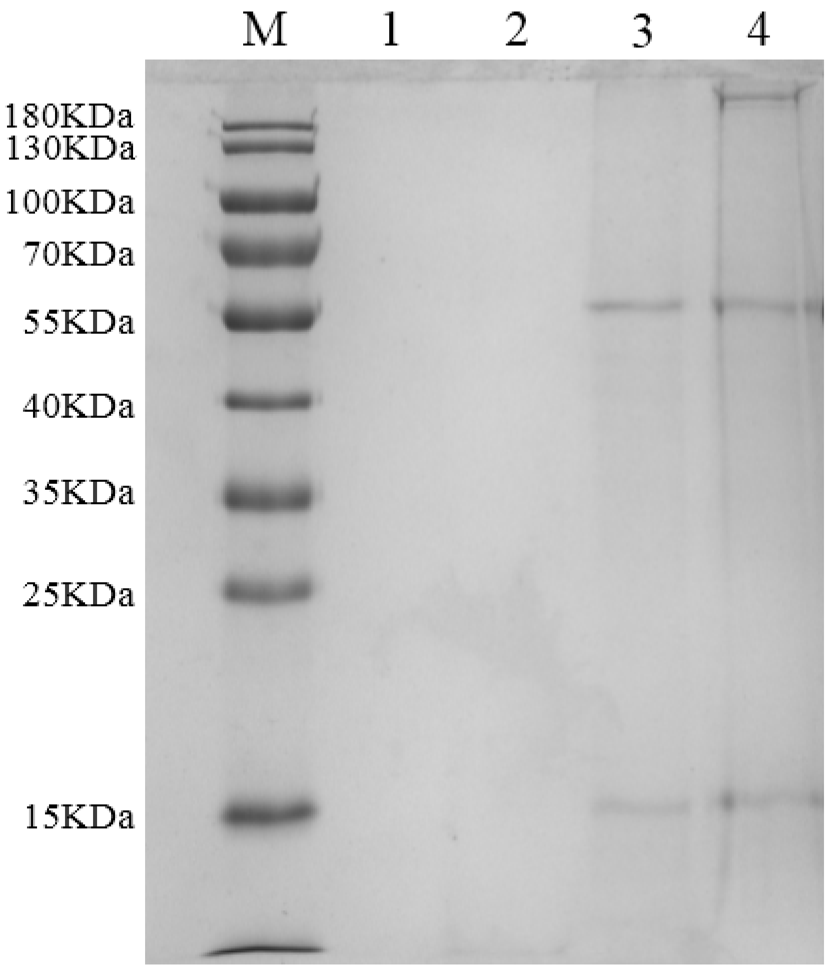

Figure S10: The result of DNA Pull down.

M：Marker 1

1: The interaction result of *Lsi1* gene promoter without the biotin labeling in Dular-WT

2: The interaction result of *Lsi1* gene promoter without the biotin labeling in Dular-OE

3: The interaction result of *Lsi1* gene promoter with the biotin labeling in Dular-WT

4: The interaction result of *Lsi1* gene promoter with the biotin labeling in Dular-OE

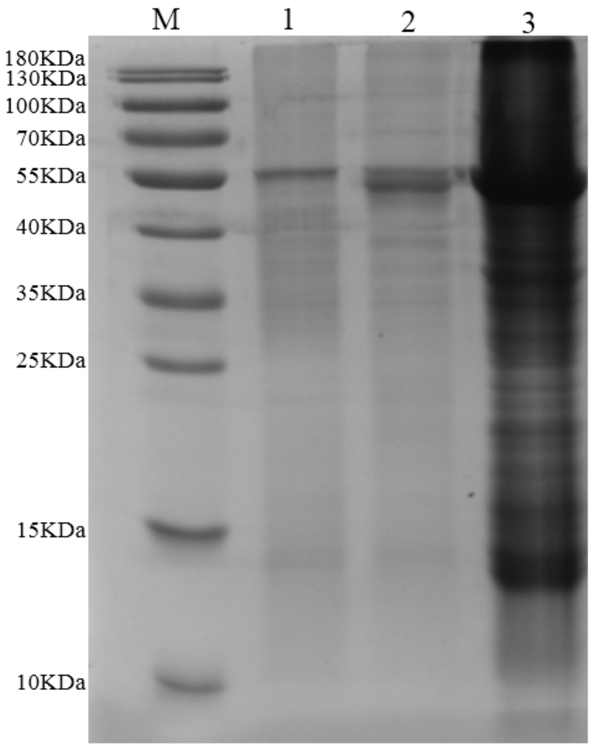

Figure S11: The interacting proteins of NIP by Co-IP.

M:mark

1: The Co-IP of Dular-OE leaf protein together with beads

2. The Co-IP of Dular-OE leaf protein together with beads and antibody

3. The leaf protein in Dular-OE

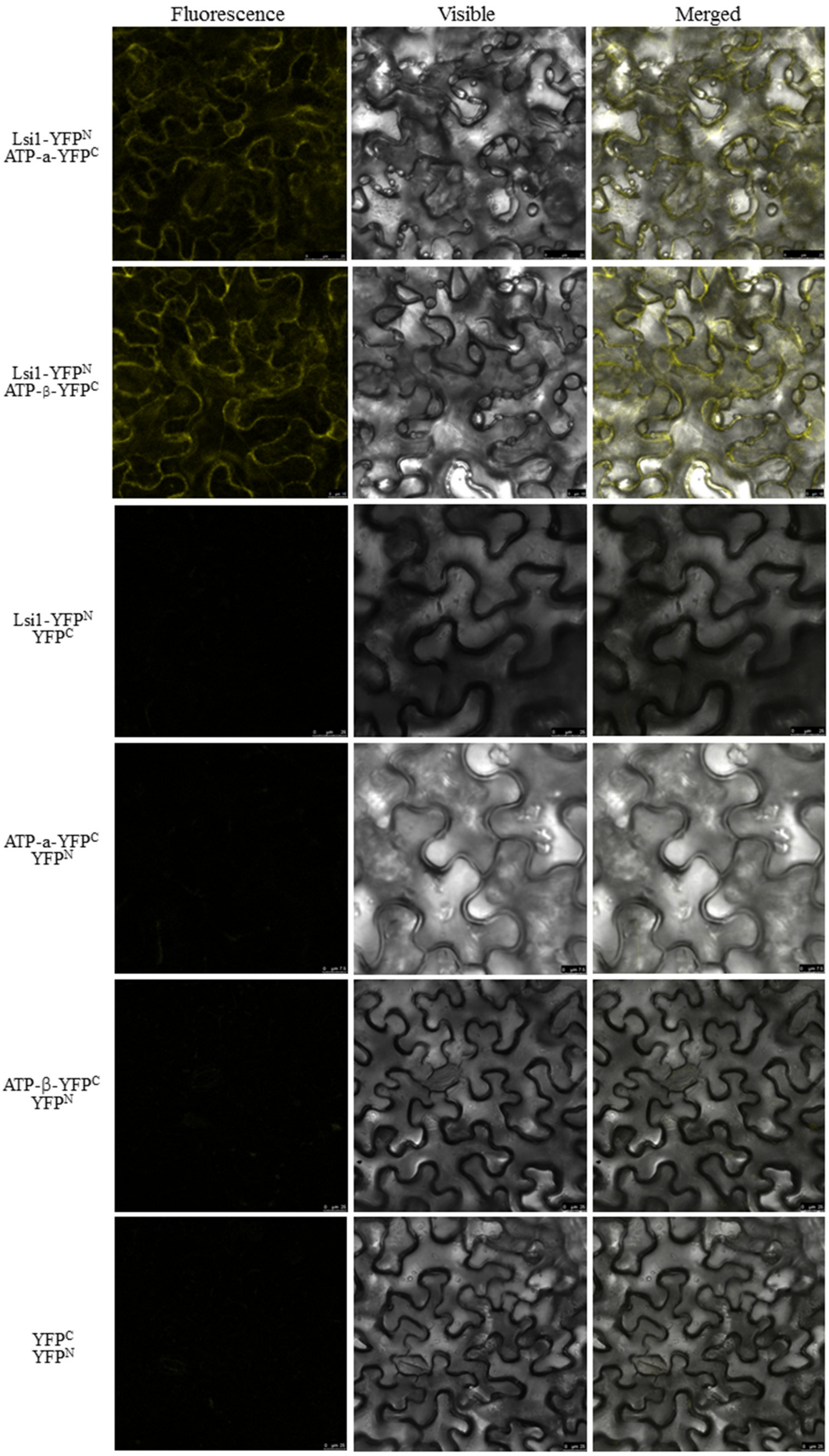

Figure S12: The confirmation of Lsi1 interactions with the ATP synthase α subunit and ATP synthase β subunit by BiFC

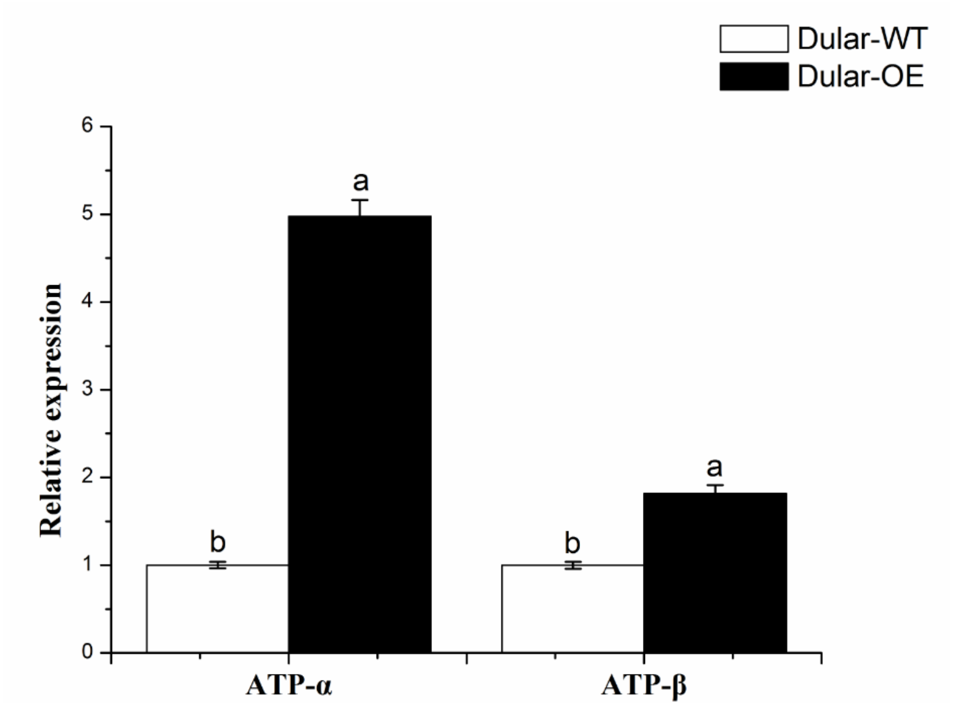

Figure S13: The verification of the gene expression levels of ATP synthase α subunit and ATP synthase β subunit by qPCR. Columns with different letters are significantly different (LSD test, $P < 0.05$). Error bars are standard error (±SE) of three biological replications.

Figure S14: Western blot verify the expression of 14-3-3f and CIPK proteins

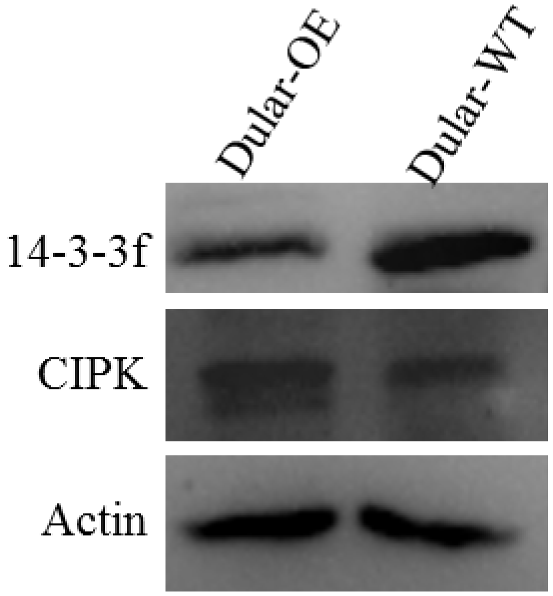

Table S1 List of leaf proteins with differential expressions between Dular-OX and Dular-WT

BinCode[a]	BinName[b]	Accession Number	Protein Name	Fold change[c]
PS				
1.1.1.1	PS.lightreaction.photosystem II.LHC-II	loc_os01g52240.1	chlorophyll a-b binding protein 2	2.04
1.1.1.1	PS.lightreaction.photosystem II.LHC-II	loc_os06g21590.1	chlorophyll a-b binding protein 6A	1.92
1.1.1.1	PS.lightreaction.photosystem II.LHC-II	loc_os07g37240.1	expressed protein	1.72
1.1.1.1	PS.lightreaction.photosystem II.LHC-II	loc_os02g10390.1	chlorophyll a-b binding protein 8	2.06
1.1.1.1	PS.lightreaction.photosystem II.LHC-II	loc_os03g39610.1	chlorophyll a-b binding protein	1.95
1.1.1.1	PS.lightreaction.photosystem II.LHC-II	loc_os07g38960.1	chlorophyll a-b binding protein	2.03
1.1.1.1	PS.lightreaction.photosystem II.LHC-II	loc_os07g37550.1	chlorophyll a-b binding protein of LHCII type III	1.85
1.1.1.1	PS.lightreaction.photosystem II.LHC-II	loc_os08g33820.1	chlorophyll a-b binding protein 4	1.74
1.1.1.1	PS.lightreaction.photosystem II.LHC-II	loc_os02g52650.1	chlorophyll a-b binding protein 4	2.07
1.1.1.1	PS.lightreaction.photosystem II.LHC-II	loc_os11g13890.1	chlorophyll a-b binding protein M9	1.85
1.1.1.1	PS.lightreaction.photosystem II.LHC-II	loc_os09g17740.1	chlorophyll a-b binding protein 1	1.80
1.1.1.1	PS.lightreaction.photosystem II.LHC-II	loc_os01g41710.1	chlorophyll a-b binding protein 2	2.77
1.1.1.1	PS.lightreaction.photosystem II.LHC-II	loc_os04g38410.1	chlorophyll a-b binding protein CP24	1.84
1.1.1.2	PS.lightreaction.photosystem II.PSII polypeptide subunits	loc_os08g10020.1	photosystem II 10 kDa polypeptide	2.13
1.1.1.2	PS.lightreaction.photosystem II.PSII polypeptide subunits	loc_os01g64960.1	photosystem II 22 kDa protein	2.03
1.1.1.2	PS.lightreaction.photosystem II.PSII polypeptide subunits	loc_os03g21560.1	photosystem II 11 kD protein	1.55
1.1.1.2	PS.lightreaction.photosystem II.PSII polypeptide subunits	loc_os03g17174.1	calcium ion binding protein	1.93
1.1.1.2	PS.lightreaction.photosystem II.PSII polypeptide	loc_os07g01480.2	oxygen evolving enhancer protein 3	1.54

	subunits		containing protein	
1.1.1.2	PS.lightreaction.photosystem II.PSII polypeptide subunits	loc_os04g16770.1	photosystem Q	1.65
1.1.1.2	PS.lightreaction.photosystem II.PSII polypeptide subunits	loc_os01g31690.1	oxygen-evolving enhancer protein 1	1.88
1.1.1.2	PS.lightreaction.photosystem II.PSII polypeptide subunits	loc_os02g36850.1	oxygen evolving enhancer protein 3	1.53
1.1.1.2	PS.lightreaction.photosystem II.PSII polypeptide subunits	loc_os08g25900.1	oxygen-evolving complex/thylakoid lumenal 25.6kDa protein	1.55
1.1.1.2	PS.lightreaction.photosystem II.PSII polypeptide subunits	loc_os12g37710.1	thylakoid lumenal 21.5 kDa protein	1.82
1.1.1.2	PS.lightreaction.photosystem II.PSII polypeptide subunits	loc_os07g36080.1	oxygen-evolving enhancer protein 3-1	2.50
1.1.2.1	PS.lightreaction.photosystem I.LHC-I	loc_os01g40710.1	OHP2	1.84
1.1.2.2	PS.lightreaction.photosystem I.PSI polypeptide subunits	loc_os12g08770.1	photosystem I reaction center subunit N	2.25
1.1.2.2	PS.lightreaction.photosystem I.PSI polypeptide subunits	loc_os09g30340.1	photosystem I reaction center subunit V	1.75
1.1.2.2	PS.lightreaction.photosystem I.PSI polypeptide subunits	loc_os08g44680.1	photosystem I reaction center subunit II	2.00
1.1.2.2	PS.lightreaction.photosystem I.PSI polypeptide subunits	loc_os03g56670.2	photosystem I reaction center subunit III	2.13
1.1.2.2	PS.lightreaction.photosystem I.PSI polypeptide subunits	loc_os12g23200.1	photosystem I reaction center subunit XI	2.68
1.1.2.2	PS.lightreaction.photosystem I.PSI polypeptide subunits	loc_os04g16760.1	photosystem I P700 chlorophyll a apoprotein A1	1.77
1.1.2.2	PS.lightreaction.photosystem I.PSI polypeptide subunits	loc_os05g48630.2	expressed protein	2.14
1.1.3	PS.lightreaction.cytochrome b6/f	loc_os07g37030.1	cytochrome b6-f complex iron-sulfur subunit	2
1.1.4	PS.lightreaction.ATP synthase	loc_os01g58000.1	ATP synthase epsilon subunit	2.16
1.1.4	PS.lightreaction.ATP synthase	loc_os03g17070.1	ATP synthase B subunit	3.05

1.1.4	PS.lightreaction.ATP synthase	loc_os07g32880.1	ATP synthase gamma subunit	2.10
1.1.4	PS.lightreaction.ATP synthase	loc_os04g16740.1	ATP synthase alpha subunit	2.11
1.1.4	PS.lightreaction.ATP synthase	loc_os02g51470.1	ATP synthase delta subunit	1.61
1.1.5.1	PS.lightreaction.other electron carrier (ox/red).plastocyanin	loc_os06g01210.1	plastocyanin	1.77
1.1.5.2	PS.lightreaction.other electron carrier (ox/red).ferredoxin	loc_os07g30670.1	electron carrier/ electron transporter/ iron ion binding protein	1.61
1.1.5.3	PS.lightreaction.other electron carrier (ox/red).ferredoxin reductase	loc_os02g01340.2	ferredoxin--NADP reductase, leaf isozyme	1.82
1.1.5.3	PS.lightreaction.other electron carrier (ox/red).ferredoxin reductase	loc_os06g01850.1	ferredoxin--NADP reductase, leaf isozyme	1.82
1.1.6	PS.lightreaction.NADH DH	loc_os01g72950.1	expressed protein	1.95
1.1.6	PS.lightreaction.NADH DH	loc_os01g66000.1	NADH dehydrogenase I subunit N	1.74
1.1.30	PS.lightreaction.state transition	loc_os05g47560.1	serine/threonine-protein kinase SNT7	1.71
1.2.1	PS.photorespiration.phosphoglycolate phosphatase	loc_os04g41340.1	4-nitrophenylphosphatase	1.82
1.2.2	PS.photorespiration.glycolate oxydase	loc_os03g57220.1	hydroxyacid oxidase 1	1.54
1.2.6	PS.photorespiration.hydroxypyruvate reductase	loc_os02g01150.2	hydroxypyruvate reductase	1.73
1.2.7	PS.photorespiration.glycerate kinase	loc_os01g48990.1	protein Kinase C630.09c	1.57
1.3.1	PS.calvin cyle.rubisco large subunit	loc_os12g10580.1	ribulose bisphosphate carboxylase large chain	2.35
1.3.2	PS.calvin cyle.rubisco small subunit	loc_os12g19381.1	ribulose bisphosphate carboxylase small chain C,	2.53
1.3.2	PS.calvin cyle.rubisco small subunit	loc_os02g05830.1	ribulose bisphosphate carboxylase small chain SSU40B	0.64
1.3.3	PS.calvin cyle.phosphoglycerate kinase	loc_os05g41640.2	phosphoglycerate kinase	1.64
1.3.4	PS.calvin cyle.GAP	loc_os03g03720.1	glyceraldehyde-3-phosphate	1.54

			dehydrogenase B	
1.3.4	PS.calvin cyle.GAP	loc_os04g38600.2	glyceraldehyde-3-phosphate dehydrogenase A	1.80
1.3.6	PS.calvin cyle.aldolase	loc_os11g07020.1	fructose-bisphosphate aldolase	1.69
1.3.7	PS.calvin cyle.FBPase	loc_os03g16050.1	fructose-1,6-bisphosphatase	1.55
1.3.9	PS.calvin cyle.seduheptulose bisphosphatase	loc_os04g16680.1	sedoheptulose-1,7-bisphosphatase	1.75
1.3.12	PS.calvin cyle.PRK	loc_os02g47020.1	phosphoribulokinase	1.78
1.3.13	PS.calvin cyle.rubisco interacting	loc_os04g56320.1	ribulose bisphosphate carboxylase/oxygenase activase	1.60
1.3.13	PS.calvin cyle.rubisco interacting	loc_os11g47970.1	ribulose bisphosphate carboxylase/oxygenase activase	1.72
Major CHO metabolism				
2.1.1.1	major CHO metabolism.synthesis.sucrose.SPS	loc_os01g69030.1	sucrose-phosphate synthase	1.78
2.1.1.3	major CHO metabolism.synthesis.sucrose.FBPase	loc_os01g64660.2	fructose-1,6-bisphosphatase	1.93
2.2.1.5	major CHO metabolism.degradation.sucrose.Susy	loc_os03g22120.1	sucrose synthase 2	0.66
2.2.2.1	major CHO metabolism.degradation.starch.starch cleavage	loc_os03g22790.1	beta-amylase	0.59
2.2.2.1	major CHO metabolism.degradation.starch.starch cleavage	loc_os08g36910.1	alpha-amylase isozyme 3D precursor	0.559
2.2.2.1	major CHO metabolism.degradation.starch.starch cleavage	loc_os07g35940.1	beta-amylase	0.61
Minor CHO metabolism				
3.4.2	minor CHO metabolism.myo-inositol.InsP-Kinases	loc_os02g32370.1	1 inositol hexaphosphate kinase	0.48
3.5	minor CHO metabolism.others	loc_os10g37330.1	aldo-keto reductase/ oxidoreductase	1.57
3.5	minor CHO metabolism.others	loc_os10g06720.1	aldose 1-epimerase	0.66
3.8.1	minor CHO metabolism.galactose.galactokinases	loc_os04g51880.1	galactokinase	0.66

Glycolysis				
4.4	glycolysis.PPFK	loc_os08g34050.1	6-phosphofructokinase 2	0.38
4.9	glycolysis.glyceraldehyde 3-phosphate dehydrogenase	loc_os06g45590.1	glyceraldehyde-3-phosphate dehydrogenase	0.64
4.9	glycolysis.glyceraldehyde 3-phosphate dehydrogenase	loc_os04g38600.2	glyceraldehyde-3-phosphate dehydrogenase A	1.80
4.9	glycolysis.glyceraldehyde 3-phosphate dehydrogenase	loc_os03g03720.1	glyceraldehyde-3-phosphate dehydrogenase B	1.54
4.14	glycolysis.PEPCase	loc_os01g11054.1	phosphoenolpyruvate carboxylase 1	1.99
Fermentation				
5.2	fermentation.PDC	loc_os05g39320.1	pyruvate decarboxylase isozyme 1	0.42
5.3	fermentation.ADH	loc_os11g10480.1	alcohol dehydrogenase 1	0.63
5.3	fermentation.ADH	loc_os03g09020.1	alcohol dehydrogenase 3	0.35
5.10	fermentation.aldehyde dehydrogenase	loc_os02g49720.3	aldehyde dehydrogenase	0.55
Gluconeogenese				
6.2	gluconeogenese/ glyoxylate cycle.malate synthase	loc_os04g40990.1	malate synthase, glyoxysomal	0.63
6.3	gluconeogenesis.Malate DH	loc_os03g56280.1	malate dehydrogenase	1.54
6.5	gluconeogenese/ glyoxylate cycle.pyruvate dikinase	loc_os05g33570.1	phosphate dikinase	1.79
6.5	gluconeogenese/ glyoxylate cycle.pyruvate dikinase	loc_os03g31750.1	phosphate dikinase	0.65
6.9	gluconeogenese/ glyoxylate cycle.isocitrate lyase	loc_os04g31700.1	methylisocitrate lyase 2	0.66
TCA				
8.3	TCA / org. transformation.carbonic anhydrases	loc_os08g32750.1	carbonic anhydrase precursor	1.75
8.3	TCA / org. transformation.carbonic anhydrases	loc_os01g45274.1	chloroplast precursor	2.35
Mitochondrial electron transport				
9.1.2	mitochondrial electron transport / ATP synthesis.NADH-DH.localisation not clear	loc_os07g39710.1	NADH-ubiquinone oxidoreductase 18 kDa subunit	0.66

Cell wall				
10.1.2	cell wall.precursor synthesis.UGE	loc_os05g51670.1	UDP-glucose 4-epimerase GEPI48	0.62
10.6.1	cell wall.degradation.cellulases and beta -1,4-glucanases	loc_os02g03120.1	endoglucanase 1 precursor	1.62
10.6.1	cell wall.degradation.cellulases and beta -1,4-glucanases	loc_os04g40510.1	hydrolase, hydrolyzing O-glycosyl compounds	0.61
10.6.3	cell wall.degradation.pectate lyases and polygalacturonases	loc_os10g26940.1	polygalacturonase-1 non-catalytic beta subunit	0.21
10.7	cell wall.modification	loc_os10g40730.1	beta-expansin 1a precursor	0.61
10.7	cell wall.modification	loc_os06g48180.1	xyloglucan endotransglucosylase/hydrolase protein 23 precursor	0.50
10.7	cell wall.modification	loc_os06g48200.1	xyloglucan endotransglucosylase/hydrolase protein 23 precursor	0.65
10.7	cell wall.modification	loc_os10g39640.1	expansin-like 3 precursor	0.59
Lipid metabolism				
11.1.8	lipid metabolism.FA synthesis and FA elongation.acyl coa ligase	loc_os04g58710.1	peroxisomal-coenzyme A synthetase	1.53
11.1.8	lipid metabolism.FA synthesis and FA elongation.acyl coa ligase	loc_os03g19250.1	AMP-binding protein	0.66
11.1.15	lipid metabolism.FA synthesis and FA elongation.ACP desaturase	loc_os01g65830.1	acyl-desaturase	0.46
11.2.4	lipid metabolism.FA desaturation.omega 6 desaturase	loc_os08g34220.1	omega-6 fatty acid desaturase	0.62
11.2.4	lipid metabolism.FA desaturation.omega 6 desaturase	loc_os02g48560.1	omega-6 fatty acid desaturase, endoplasmic reticulum isozyme 2	2.27

11.3	lipid metabolism.Phospholipid synthesis	loc_os05g47540.1	phosphoethanolamine N-methyltransferase	1.74
11.6	lipid metabolism.lipid transfer proteins etc	loc_os12g02310.1	nonspecific lipid-transfer protein 2 precursor	0.37
11.8	lipid metabolism.'exotics' (steroids, squalene etc)	loc_os02g52720.1	peroxisomal multifunctional enzyme type 2	0.64
11.8	lipid metabolism.'exotics' (steroids, squalene etc)	loc_os09g08190.1	flavonol 4-sulfotransferase	3.16
11.8.10	lipid metabolism.'exotics' (steroids, squalene etc).phosphatidylcholinesterol O-acyltransferase	loc_os02g37654.1	1-O-acylceramide synthase precursor	2.41
11.9.2.1	lipid metabolism.lipid degradation.lipases.triacylglycerol lipase	loc_os04g56240.1	triacylglycerol lipase	1.59
11.9.2.1	lipid metabolism.lipid degradation.lipases.triacylglycerol lipase	loc_os05g06140.1	lipase	0.37
11.9.3.3	lipid metabolism.lipid degradation.lysophospholipases.glycerophosphodiester phosphodiesterase	loc_os02g37590.1	glycerophosphoryl diester phosphodiesterase family protein	2.01
N-metabolism				
12.1.1	N-metabolism.nitrate metabolism.NR	loc_os08g36480.1	nitrate reductase 1	1.83
12.1.2	N-metabolism.nitrate metabolism.nitrite reductase	loc_os01g25484.1	ferredoxin--nitrite reductase	1.65
12.2.2	N-metabolism.ammonia metabolism.glutamine synthase	loc_os04g56400.1	glutamine synthetase	1.58
12.2.99	N-metabolism.ammonia metabolism.unspecified	loc_os08g14580.1	haloacid dehalogenase-like hydrolase domain-containing protein 1A	0.62
Amino acid metabolism				
13.1.3.1	amino acid metabolism.synthesis.aspartate family.asparagine	loc_os03g18130.1	asparagine synthetase	0.55
13.1.3.6.1.1	amino acid metabolism.synthesis.aspartate family.misc.homoserine.aspartate kinase	loc_os12g03190.1	bifunctional aspartokinase/homoserine dehydrogenase I	4.17

13.1.5.3.1	amino acid metabolism.synthesis.serine-glycine-cysteine group.cysteine.OASTL	loc_os01g59920.1	cysteine synthase, chloroplast precursor	1.60
13.1.7.6	amino acid metabolism.synthesis.histidine.histidinol-phosphate aminotransferase	loc_os02g47940.1	histidinol-phosphate aminotransferase	0.65
13.2.3.1.1	amino acid metabolism.degradation.aspartate family.asparagine.L-asparaginase	loc_os04g55710.1	transposon protein, putative	3.92
13.2.3.2	amino acid metabolism.degradation.aspartate family.threonine	loc_os02g17920.1	lactoylglutathione lyase	1.60
13.2.5.1	amino acid metabolism.degradation.serine-glycine-cysteine group.serine	loc_os02g01150.2	hydroxypyruvate reductase	1.73
13.2.5.2	amino acid metabolism.degradation.serine-glycine-cysteine group.glycine	loc_os01g12830.2	glyoxylate reductase	1.59
13.2.5.2	amino acid metabolism.degradation.serine-glycine-cysteine group.glycine	loc_os02g01150.2	hydroxypyruvate reductase	1.73
13.2.5.2	amino acid metabolism.degradation.serine-glycine-cysteine group.glycine	loc_os06g40940.1	glycine dehydrogenase 2	1.62
S-assimilation				
14.2	S-assimilation.APR	loc_os07g32570.1	OsAPRL1 - Oryza sativa adenosine 5'-phosphosulfate reductase-like	2.38
Metal handling				

15.1	metal handling.acquisition	loc_os04g36720.1	ferric reductase-like transmembrane component	1.93
Secondary metabolism				
16.1.1.10	secondary metabolism.isoprenoids.non-mevalonate pathway.geranylgeranyl pyrophosphate synthase	loc_os06g46450.1	decaprenyl-diphosphate synthase subunit 1	0.61
16.1.2.9	secondary metabolism.isoprenoids.mevalonate pathway.farnesyl pyrophosphate synthetase	loc_os04g56230.1	farnesyl pyrophosphate synthetase	1.97
16.1.4	secondary metabolism.isoprenoids.carotenoids	loc_os07g43370.1	oxidoreductase	1.56
16.1.5	secondary metabolism.isoprenoids.terpenoids	loc_os11g18366.2	cycloartenol synthase	1.53
16.1.5	secondary metabolism.isoprenoids.terpenoids	loc_os03g22620.1	terpene synthase 7	4.47
16.1.5	secondary metabolism.isoprenoids.terpenoids	loc_os04g27430.1	terpene synthase 7	0.49
16.2	secondary metabolism.phenylpropanoids	loc_os04g11970.1	O-methyltransferase ZRP4, putative	0.64
16.2	secondary metabolism.phenylpropanoids	loc_os11g20160.1	12011.m06091 protein O-methyltransferase ZRP4	0.60
16.2	secondary metabolism.phenylpropanoids	loc_os11g07960.1	anthranilate N-benzoyltransferase protein 1	0.52
16.2	secondary metabolism.phenylpropanoids	loc_os11g20090.1	O-methyltransferase ZRP4	0.49
16.2	secondary metabolism.phenylpropanoids	loc_os11g19840.1	O-methyltransferase ZRP4	1.57
16.2	secondary metabolism.phenylpropanoids	loc_os12g25450.1	O-methyltransferase ZRP4	1.99
16.2.1.1	secondary metabolism.phenylpropanoids.lignin biosynthesis.PAL	loc_os04g43800.1	phenylalanine ammonia-lyase	1.97
16.2.1.1	secondary metabolism.phenylpropanoids.lignin biosynthesis.PAL	loc_os02g41650.3	phenylalanine ammonia-lyase	1.82
16.2.1.10	secondary metabolism.phenylpropanoids.lignin biosynthesis.CAD	loc_os09g23530.1	mannitol dehydrogenase	0.54
16.2.1.10	secondary metabolism.phenylpropanoids.lignin	loc_os09g23550.1	mannitol dehydrogenase	1.51

	biosynthesis.CAD			
16.2.1.10	secondary metabolism.phenylpropanoids.lignin biosynthesis.CAD	loc_os09g23540.1	mannitol dehydrogenase	2.07
16.4.1	secondary metabolism.N misc.alkaloid-like	loc_os06g35700.1	reticuline oxidase precursor	0.45
16.4.1	secondary metabolism.N misc.alkaloid-like	loc_os03g16230.1	tropinone reductase	2.06
16.8.2	secondary metabolism.flavonoids.chalcones	loc_os03g60509.2	expressed protein	3.32
16.8.2	secondary metabolism.flavonoids.chalcones	loc_os11g35930.1	bibenzyl synthase	0.48
16.8.3	secondary metabolism.flavonoids.dihydroflavonols	loc_os09g31490.1	dihydroflavonol-4-reductase	4.00
16.8.3	secondary metabolism.flavonoids.dihydroflavonols	loc_os08g34280.1	dihydroflavonol-4-reductase	2.11
16.8.3	secondary metabolism.flavonoids.dihydroflavonols	loc_os04g53810.1	leucoanthocyanidin reductase	1.97
16.8.3	secondary metabolism.flavonoids.dihydroflavonols	loc_os09g25150.1	dihydroflavonol-4-reductase	2.11
16.8.5	secondary metabolism.flavonoids.isoflavonols	loc_os01g01650.1	isoflavone reductase homolog IRL	3.66
16.8.5	secondary metabolism.flavonoids.isoflavonols	loc_os12g16240.1	protein isoflavone reductase	1.92
16.8.5	secondary metabolism.flavonoids.isoflavonols	loc_os12g16220.2	isoflavone reductase	0.58
Hormone metabolism				
17.1.1	hormone metabolism.abscisic acid.synthesis-degradation	loc_os03g57680.1	aldehyde oxidase 1	1.61
17.2.1	hormone metabolism.auxin.synthesis-degradation	loc_os03g62060.1	IAA-amino acid hydrolase ILR1 precursor	0.49
17.2.3	hormone metabolism.auxin.induced-regulated-responsive-activated	loc_os04g27060.1	auxin-induced protein PCNT115	0.35
17.2.3	hormone metabolism.auxin.induced-regulated-responsive-activated	loc_os01g48850.1	dopamine beta-monooxygenase	0.51
17.2.3	hormone metabolism.auxin.induced-regulated-responsive-activat	loc_os04g58280.2	stem-specific protein TSJT1	0.45

	ed			
17.3.1.2.7	hormone metabolism.brassinosteroid.synthesis-degradation.sterols.DWF5	loc_os02g26650.3	7-dehydrocholesterol reductase	0.47
17.5.1	hormone metabolism.ethylene.synthesis-degradation	loc_os01g27490.1	leucoanthocyanidin dioxygenase	0.53
17.5.1	hormone metabolism.ethylene.synthesis-degradation	loc_os03g32470.1	leucoanthocyanidin dioxygenase	0.49
17.5.3	hormone metabolism.ethylene.induced-regulated-responsive-activated	loc_os10g01080.1	pyridoxin biosynthesis protein ER1	1.57
17.7.1.2	hormone metabolism.jasmonate.synthesis-degradation.lipoxygenase	loc_os08g39840.1	lipoxygenase 7	0.66
17.7.1.2	hormone metabolism.jasmonate.synthesis-degradation.lipoxygenase	loc_os02g10120.1	lipoxygenase 2.3	2.11
17.7.1.3	hormone metabolism.jasmonate.synthesis-degradation.allene oxidase synthase	loc_os03g12500.1	cytochrome P450 74A2	0.45
17.7.1.3	hormone metabolism.jasmonate.synthesis-degradation.allene oxidase synthase	loc_os02g02000.1	cytochrome P450 74A4	1.57
17.7.1.5	hormone metabolism.jasmonate.synthesis-degradation.12-Oxo-PDA-reductase	loc_os06g11210.1	12-oxophytodienoate reductase 2	0.58
Co-factor and vitamine metabolism				
18	Co-factor and vitamine metabolism	loc_os10g01080.1	pyridoxin biosynthesis protein ER1	1.57

Tetrapyrrole synthesis				
19.40	tetrapyrrole synthesis.regulation	loc_os11g16550.1	expressed protein	1.52
19.40	tetrapyrrole synthesis.regulation	loc_os01g32730.1	FLU	1.71
Stress				
20.1	stress.biotic	loc_os10g39680.1	acidic endochitinase Q precursor	0.27
20.1	stress.biotic	loc_os02g10350.1	MLO-like protein 2	1.71
20.1	stress.biotic	loc_os07g03368.1	pathogenesis-related protein PRB1-3 precursor	0.34
20.1	stress.biotic	loc_os02g34490.1	Leucine Rich Repeat family protein	1.74
20.1	stress.biotic	loc_os11g42450.1	protein binding protein	0.66
20.1	stress.biotic	loc_os07g03467.1	pathogenesis-related protein PRB1-3 precursor	0.42
20.1	stress.biotic	loc_os11g11960.1	protein NBS-LRR type disease resistance protein	0.59
20.1	stress.biotic	loc_os04g41680.1	endochitinase A precursor	0.53
20.1	stress.biotic	loc_os12g38150.1	osmotin-like protein precursor	0.28
20.1	stress.biotic	loc_os08g05440.1	NB-ARC domain containing protein	0.50
20.1	stress.biotic	loc_os05g31530.1	disease resistance protein RGA4	1.62
20.1	stress.biotic	loc_os08g40690.1	xylanase inhibitor protein 1 precursor	0.63
20.1	stress.biotic	loc_os11g47550.1	xylanase inhibitor protein 2 precursor	0.58
20.1	stress.biotic	loc_os10g34930.1	secretory protein	0.34
20.1	stress.biotic	loc_os12g43410.1	thaumatin-like protein precursor	0.62
20.1	stress.biotic	loc_os03g46070.1	protein P21, putative	0.56
20.1	stress.biotic	loc_os04g09390.1	lectin precursor	0.56
20.1	stress.biotic	loc_os03g46060.1	osmotin-like protein OSML13 precursor	0.60
20.1	stress.biotic	loc_os07g01530.1	expressed protein	1.56

20.1	stress.biotic	loc_os01g49320.1	acidic endochitinase precursor	0.34
20.1	stress.biotic	loc_os06g51060.1	basic endochitinase 1 precursor	0.66
20.1	stress.biotic	loc_os11g47500.1	xylanase inhibitor protein 1 precursor	0.37
20.1	stress.biotic	loc_os03g45960.1	protein P21	0.64
20.1	stress.biotic	loc_os12g43450.1	pathogenesis-related protein precursor	1.54
20.1	stress.biotic	loc_os10g11500.1	pathogenesis-related protein PRB1-3 precursor	0.38
20.1	stress.biotic	loc_os10g34920.1	secretory protein	0.59
20.1	stress.biotic	loc_os11g31540.1	BRASSINOSTEROID INSENSITIVE 1-associated receptor kinase 1 precursor	0.60
20.1	stress.biotic	loc_os10g34910.1	secretory protein	0.61
20.1	stress.biotic	loc_os10g42190.1	protein lap4	0.66
20.1	stress.biotic	loc_os07g01620.1	dirigent-like protein pDIR12	0.62
20.1	stress.biotic	loc_os04g41620.1	endochitinase A precursor	0.33
20.1	stress.biotic	loc_os01g28500.1	pathogenesis-related protein PRMS precursor	0.66
20.1	stress.biotic	loc_os12g38170.1	pathogenesis-related protein 5 precursor	2.75
20.1	stress.biotic	loc_os08g09110.1	NB-ARC domain containing protein	1.57
20.1	stress.biotic	loc_os05g15770.1	xylanase inhibitor protein 2 precursor	0.51
20.1	stress.biotic	loc_os07g03710.1	1 pathogenesis-related protein PRB1-3 precursor	0.42
20.1	stress.biotic	loc_os07g03580.1	pathogenesis-related protein PRB1-2 precursor	0.38
20.2	stress.abiotic	loc_os06g02470.1	ATOZI1	1.57
20.2	stress.abiotic	loc_os12g26290.1	alpha-DOX2	1.74
20.2	stress.abiotic	loc_os05g13330.1	CAB2	0.66

20.2.1	stress.abiotic.heat	loc_os02g30320.1	drought-induced protein 1	1.53
20.2.1	stress.abiotic.heat	loc_os06g11610.1	heat shock 22 kDa protein	0.40
20.2.1	stress.abiotic.heat	loc_os03g58790.1	ATPase 3	0.55
20.2.1	stress.abiotic.heat	loc_os12g31460.1	heat shock protein binding protein	1.73
20.2.1	stress.abiotic.heat	loc_os03g14040.1	expressed protein	1.73
20.2.1	stress.abiotic.heat	loc_os02g52150.2	heat shock 22 kDa protein	0.59
20.2.2	stress.abiotic.cold	loc_os09g39770.1	C2 domain containing protein	0.61
20.2.3	stress.abiotic.drought/salt	loc_os01g13210.1	DREPP4 protein	1.67
20.2.5	stress.abiotic.light	loc_os10g01044.1	tic62 protein	1.92
20.2.99	stress.abiotic.unspecified	loc_os01g19820.2	universal stress protein	0.59
20.2.99	stress.abiotic.unspecified	loc_os06g02470.1	ATOZI1	1.57
20.2.99	stress.abiotic.unspecified	loc_os10g30150.1	ethylene-responsive protein	0.51
20.2.99	stress.abiotic.unspecified	loc_os03g58980.1	germin-like protein subfamily T member 2 precursor	0.62
20.2.99	stress.abiotic.unspecified	loc_os02g47840.1	ethylene response protein	0.59
20.2.99	stress.abiotic.unspecified	loc_os08g08970.1	germin-like protein subfamily 1 member 11 precursor	0.51
20.2.99	stress.abiotic.unspecified	loc_os01g14670.1	nectarin-1 precursor, putative, expressed	0.65
20.2.99	stress.abiotic.unspecified	loc_os05g28740.1	universal stress protein	0.51
20.2.99	stress.abiotic.unspecified	loc_os08g08980.1	germin-like protein subfamily 1 member 7 precursor	0.62
20.2.99	stress.abiotic.unspecified	loc_os03g19270.1	ER6 protein	0.30
20.2.99	stress.abiotic.unspecified	loc_os12g36640.1	fiber protein Fb19	1.59
20.2.99	stress.abiotic.unspecified	loc_os07g47620.1	universal stress protein	0.41
Redox				
21.1	redox.thioredoxin	loc_os12g08730.1	thioredoxin M-type, chloroplast precursor	1.64

21.1	redox.thioredoxin	loc_os03g55820.1	thiol disulfide interchange protein txlA	1.59
21.1	redox.thioredoxin	loc_os02g42700.1	thioredoxin M-type	0.55
21.1	redox.thioredoxin	loc_os11g32500.1	rubredoxin family protein	1.73
21.1	redox.thioredoxin	loc_os04g44650.1	ferredoxin-thioredoxin reductase	0.55
21.2.1	redox.ascorbate and glutathione.ascorbate	loc_os06g37150.1	L-ascorbate oxidase precursor	1.93
21.2.2	redox.ascorbate and glutathione.glutathione	loc_os04g46960.2	phospholipid hydroperoxide glutathione peroxidase	0.53
21.3	redox.heme	loc_os03g13140.1	non-symbiotic hemoglobin 2	0.51
21.4	redox.glutaredoxins	loc_os01g34620.1	OsGrx_S15.1 - glutaredoxin subgroup II	0.64
21.4	redox.glutaredoxins	loc_os06g44910.1	OsGrx_C4 - glutaredoxin subgroup I	0.64
21.4	redox.glutaredoxins	loc_os01g13480.1	electron transporter/ thiol-disulfide exchange intermediate	1.83
21.5	redox.peroxiredoxin	loc_os07g44430.1	peroxiredoxin	0.56
21.5	redox.peroxiredoxin	loc_os06g09610.1	peroxiredoxin bcp	1.58
21.5	redox.peroxiredoxin	loc_os02g09940.1	peroxiredoxin-5, mitochondrial precursor	1.51
Nucleotide metabolism.synthesis				
23.1.2.1	nucleotide metabolism.synthesis.purine.amidophosphoribosyltransferase	loc_os01g65260.1	amidophosphoribosyltransferase	1.67
23.2	nucleotide metabolism.degradation	loc_os05g33630.4	hydrolase	0.64
23.2	nucleotide metabolism.degradation	loc_os09g39440.1	pyrimidine-specific ribonucleoside hydrolase rihB	2.22
23.2	nucleotide metabolism.degradation	loc_os05g28180.1	AMP deaminase 2	0.63
Misc				
26.1	misc.misc2	loc_os10g35540.1	epoxide hydrolase 2	0.59
26.2	misc.UDP glucosyl and glucoronyl transferases	loc_os05g45150.1	anthocyanidin 5,3-O-glucosyltransferase	0.60

26.2	misc.UDP glucosyl and glucoronyl transferases	loc_os07g10190.1	anthocyanidin 3-O-glucosyltransferase	1.69
26.2	misc.UDP glucosyl and glucoronyl transferases	loc_os07g10160.1	UDP-rhamnose rhamnosyltransferase	0.34
26.2	misc.UDP glucosyl and glucoronyl transferases	loc_os07g32060.1	anthocyanidin 5,3-O-glucosyltransferase	2.53
26.2	misc.UDP glucosyl and glucoronyl transferases	loc_os02g11110.1	flavonol-3-O-glycoside-7-O-glucosyltransferase 1	1.58
26.2	misc.UDP glucosyl and glucoronyl transferases	loc_os06g18670.1	anthocyanidin 3-O-glucosyltransferase	0.63
26.2	misc.UDP glucosyl and glucoronyl transferases	loc_os06g18010.1	hydroquinone glucosyltransferase	0.48
26.2	misc.UDP glucosyl and glucoronyl transferases	loc_os01g41430.1	flavonol-3-O-glycoside-7-O-glucosyltransferase 1	0.51
26.2	misc.UDP glucosyl and glucoronyl transferases	loc_os12g37510.1	transferase, transferring glycosyl groups	0.54
26.2	misc.UDP glucosyl and glucoronyl transferases	loc_os08g38740.1	transferase, transferring glycosyl groups	2.39
26.2	misc.UDP glucosyl and glucoronyl transferases	loc_os02g37690.1	anthocyanidin 3-O-glucosyltransferase	4.08
26.2	misc.UDP glucosyl and glucoronyl transferases	loc_os09g34230.1	indole-3-acetate beta-glucosyltransferase	1.65
26.2	misc.UDP glucosyl and glucoronyl transferases	loc_os06g18790.1	anthocyanidin 3-O-glucosyltransferase	0.66
26.2	misc.UDP glucosyl and glucoronyl transferases	loc_os07g30620.1	cytokinin-O-glucosyltransferase 2	0.54
26.3	misc.gluco-, galacto- and mannosidases	loc_os03g49600.1	non-cyanogenic beta-glucosidase precursor	0.66
26.3	misc.gluco-, galacto- and mannosidases	loc_os02g03120.1	endoglucanase 1 precursor	1.62
26.3	misc.gluco-, galacto- and mannosidases	loc_os04g39900.1	non-cyanogenic beta-glucosidase precursor, putative, expressed	0.37
26.3	misc.gluco-, galacto- and mannosidases	loc_os09g31430.1	non-cyanogenic beta-glucosidase precursor	0.64
26.3	misc.gluco-, galacto- and mannosidases	loc_os04g40510.1	hydrolase, hydrolyzing O-glycosyl compounds	0.61
26.4	misc.beta 1,3 glucan hydrolases	loc_os02g04670.1	glucan endo-1,3-beta-glucosidase 6 precursor	0.66
26.4	misc.beta 1,3 glucan hydrolases	loc_os01g51570.1	glucan endo-1,3-beta-glucosidase GII precursor	0.49

26.4	misc.beta 1,3 glucan hydrolases	loc_os01g71830.1	glucan endo-1,3-beta-glucosidase GV	0.41
26.4	misc.beta 1,3 glucan hydrolases	loc_os07g35480.2	glucan endo-1,3-beta-glucosidase 3 precursor	0.55
26.4	misc.beta 1,3 glucan hydrolases	loc_os01g71860.1	glycosyl hydrolases family 17 protein	0.57
26.4	misc.beta 1,3 glucan hydrolases	loc_os01g71380.1	glucan endo-1,3-beta-glucosidase GII precursor	0.61
26.4	misc.beta 1,3 glucan hydrolases	loc_os05g31140.1	lichenase-2 precursor	0.62
26.7	misc.oxidases - copper, flavone etc.	loc_os04g41960.1	NADP-dependent oxidoreductase P1	0.65
26.7	misc.oxidases - copper, flavone etc.	loc_os08g29170.1	quinone oxidoreductase-like protein At1g23740	1.93
26.8	misc.nitrilases, *nitrile lyases, berberine bridge enzymes, reticuline oxidases, troponine reductases	loc_os04g55730.1	alpha-N-acetylglucosaminidase precursor	1.74
26.8	misc.nitrilases, *nitrile lyases, berberine bridge enzymes, reticuline oxidases, troponine reductases	loc_os06g35700.1	reticuline oxidase precursor	0.45
26.8	misc.nitrilases, *nitrile lyases, berberine bridge enzymes, reticuline oxidases, troponine reductases	loc_os03g16230.1	tropinone reductase	2.06
26.8	misc.nitrilases, *nitrile lyases, berberine bridge enzymes, reticuline oxidases, troponine reductases	loc_os01g70850.1	esterase PIR7B	0.61
26.9	misc.glutathione S transferases	loc_os07g28480.1	glutathione S-transferase	1.52
26.9	misc.glutathione S transferases	loc_os07g07320.1	glutathione S-transferase GSTU6	0.43
26.9	misc.glutathione S transferases	loc_os03g57200.1	glutathione S-transferase parA	0.57
26.9	misc.glutathione S transferases	loc_os01g55830.1	glutathione S-transferase GSTF2	0.66
26.9	misc.glutathione S transferases	loc_os10g38360.1	glutathione S-transferase GSTU6	0.60
26.9	misc.glutathione S transferases	loc_os10g38740.1	glutathione S-transferase GSTU6	0.54
26.9	misc.glutathione S transferases	loc_os09g29200.1	glutathione S-transferase	0.59
26.9	misc.glutathione S transferases	loc_os10g38710.1	glutathione S-transferase GSTU6	1.59

26.9	misc.glutathione S transferases	loc_os01g49720.1	glutathione S-transferase GSTU6	0.56
26.9	misc.glutathione S transferases	loc_os10g25590.1	glutathione S-transferase BZ2	0.29
26.10	misc.cytochrome P450	loc_os06g19070.1	cytochrome P450 76C2	0.66
26.10	misc.cytochrome P450	loc_os08g43440.1	flavonoid 3-monooxygenase	1.67
26.12	misc.peroxidases	loc_os02g14440.1	peroxidase 68 precursor	1.93
26.12	misc.peroxidases	loc_os01g73200.1	peroxidase 12 precursor	0.41
26.12	misc.peroxidases	loc_os06g35520.1	peroxidase 52 precursor	0.56
26.12	misc.peroxidases	loc_os01g22230.1	peroxidase 1 precursor	0.64
26.12	misc.peroxidases	loc_os01g22352.1	peroxidase 2 precursor	0.44
26.12	misc.peroxidases	loc_os04g59150.1	peroxidase 12 precursor	0.57
26.12	misc.peroxidases	loc_os04g59190.1	peroxidase 2 precursor	0.63
26.12	misc.peroxidases	loc_os01g22370.1	peroxidase 1 precursor	0.54
26.12	misc.peroxidases	loc_os04g59200.1	peroxidase 12 precursor	0.36
26.12	misc.peroxidases	loc_os01g28030.1	peroxidase 24 precursor	1.85
26.12	misc.peroxidases	loc_os02g58720.2	peroxidase 64 precursor	0.66
26.12	misc.peroxidases	loc_os07g48040.1	peroxidase 2 precursor	1.69
26.12	misc.peroxidases	loc_os01g73170.1	peroxidase 12 precursor	0.49
26.13	misc.acid and other phosphatases	loc_os05g09704.1	stem 28 kDa glycoprotein precursor	0.60
26.13	misc.acid and other phosphatases	loc_os05g10210.1	stem 28 kDa glycoprotein precursor	0.42
26.13	misc.acid and other phosphatases	loc_os05g10310.2	acid phosphatase, putative	0.59
26.16	misc.myrosinases-lectin-jacalin	loc_os11g06570.1	transposon protein, putative, CACTA, En/Spm sub-class	0.34
26.17	misc.dynamin	loc_os09g39960.1	dynamin-related protein 1C	0.59
26.19	misc.plastocyanin-like	loc_os08g37660.1	blue copper protein precursor	0.27
26.21	misc.protease inhibitor/seed storage/lipid transfer protein (LTP) family protein	loc_os05g41030.1	lipid binding protein	0.50

26.21	misc.protease inhibitor/seed storage/lipid transfer protein (LTP) family protein	loc_os06g49770.1	lipid binding protein	0.66
26.22	misc.short chain dehydrogenase/reductase (SDR)	loc_os10g31780.1	versicolorin reductase	0.63
26.22	misc.short chain dehydrogenase/reductase (SDR)	loc_os07g46830.1	sex determination protein tasselseed-2	0.63
26.22	misc.short chain dehydrogenase/reductase (SDR)	loc_os09g06499.1	sulfate transporter 4.1	1.55
26.22	misc.short chain dehydrogenase/reductase (SDR)	loc_os07g46852.1	sex determination protein tasselseed-2	0.55
26.23	misc.rhodanese	loc_os09g36040.1	rhodanese-like domain containing protein	1.64
26.23	misc.rhodanese	loc_os09g10750.1	rhodanese family protein	1.57
26.24	misc.GCN5-related N-acetyltransferase	loc_os01g42470.2	pre-mRNA-splicing factor cwc24	0.45
26.26.1	misc.aminotransferases.aminotransferase class IV family protein	loc_os01g13690.2	aldehyde dehydrogenase	2.22
26.28	misc.GDSL-motif lipase	loc_os05g11910.1	esterase precursor	0.61
26.28	misc.GDSL-motif lipase	loc_os01g11710.1	esterase precursor	0.63
26.28	misc.GDSL-motif lipase	loc_os01g11730.1	esterase precursor	0.61
26.28	misc.GDSL-motif lipase	loc_os03g62740.1	esterase precursor	0.57
26.28	misc.GDSL-motif lipase	loc_os06g06290.1	alpha-L-fucosidase 2 precursor	1.51
26.28	misc.GDSL-motif lipase	loc_os01g11620.1	esterase precursor	0.66
26.30	misc. other Ferredoxins and Rieske domain	loc_os11g13850.2	rieske domain containing protein	1.56
RNA				
27.1	RNA.processing	loc_os11g43620.1	small nuclear ribonucleoprotein F	0.20
27.1.1	RNA.processing.splicing	loc_os11g41820.1	splicing factor U2AF 65 kDa subunit	0.64
27.1.19	RNA.processing.ribonucleases	loc_os09g36700.1	extracellular ribonuclease LE precursor	0.57
27.1.19	RNA.processing.ribonucleases	loc_os09g36680.1	ribonuclease 3 precursor	0.57
27.2	RNA.transcription	loc_os10g02544.1	retrotransposon protein, putative, Ty3-gypsy subclass	1.61
27.3.23	RNA.regulation of transcription.HSF,Heat-shock	loc_os03g58790.1	ATPase 3	0.55

	transcription factor family			
27.3.30	RNA.regulation of transcription.Trihelix, Triple-Helix transcription factor family	loc_os01g21590.1	expressed protein	0.52
27.3.30	RNA.regulation of transcription.Trihelix, Triple-Helix transcription factor family	loc_os10g41460.1	expressed protein	1.51
27.3.50	RNA.regulation of transcription.General Transcription	loc_os03g01910.1	transcription factor BTF3	0.59
27.3.55	RNA.regulation of transcription.HDA	loc_os12g08220.1	histone deacetylase 10	1.52
27.3.60	RNA.regulation of transcription.NIN-like bZIP-related family	loc_os11g16290.1	NIN-like protein 2	0.49
27.3.67	RNA.regulation of transcription.putative transcription regulator	loc_os03g22730.1	nucleolar protein NOP5	0.47
27.3.67	RNA.regulation of transcription.putative transcription regulator	loc_os01g32720.1	expressed protein	0.62
27.3.67	RNA.regulation of transcription.putative transcription regulator	loc_os03g01910.1	transcription factor BTF3	0.59
27.3.99	RNA.regulation of transcription.unclassified	loc_os10g36000.1	remorin	2.11
27.3.99	RNA.regulation of transcription.unclassified	loc_os01g51870.1	S-adenosylmethionine-dependent methyltransferase,	0.55
27.3.99	RNA.regulation of transcription.unclassified	loc_os07g11110.1	mRNA binding protein precursor	1.56
27.3.99	RNA.regulation of transcription.unclassified	loc_os06g02780.1	aspartic proteinase nepenthesin-2 precursor	2.00
27.4	RNA.RNA binding	loc_os03g17010.1	THO complex subunit 4	0.64
27.4	RNA.RNA binding	loc_os08g42980.1	RNA binding protein	0.60
27.4	RNA.RNA binding	loc_os10g02630.1	U1 snRNP 70K protein	0.60
27.4	RNA.RNA binding	loc_os01g52390.1	plasminogen activator inhibitor 1 RNA-binding protein	0.63

27.4	RNA.RNA binding	loc_os05g51180.1	plasminogen activator inhibitor 1 RNA-binding protein	0.50
DNA				
28.1	DNA.synthesis/chromatin structure	loc_os01g55880.1	hemimethylated DNA binding domain containing protein	1.55
28.1.3	DNA.synthesis/chromatin structure.histone	loc_os12g25120.1	histone H2A.2	0.64
28.1.3	DNA.synthesis/chromatin structure.histone	loc_os01g32730.1	FLU	1.71
28.99	DNA.unspecified	loc_os08g23100.2	cyclase	0.58
28.99	DNA.unspecified	loc_os09g02270.1	cyclase	0.45
Protein				
29.2.1.1	protein.synthesis.ribosomal protein.prokaryotic	loc_os03g10060.1	chloroplast 30S ribosomal protein S10	2.51
29.2.1.1	protein.synthesis.ribosomal protein.prokaryotic	loc_os03g03360.1	50S ribosomal protein L5	1.66
29.2.1.1	protein.synthesis.ribosomal protein.prokaryotic	loc_os05g09400.3	PSRP4	1.59
29.2.1.1	protein.synthesis.ribosomal protein.prokaryotic	loc_os05g01110.1	50S ribosomal protein L28	2.01
29.2.1.1	protein.synthesis.ribosomal protein.prokaryotic	loc_os05g49320.1	50S ribosomal protein L12-1	2.04
29.2.1.1	protein.synthesis.ribosomal protein.prokaryotic	loc_os03g20100.1	30S ribosomal protein S1	1.99
29.2.1.1	protein.synthesis.ribosomal protein.prokaryotic	loc_os04g38750.1	plastid-specific 30S ribosomal protein 3	1.87
29.2.1.1	protein.synthesis.ribosomal protein.prokaryotic	loc_os03g34040.1	30S ribosomal protein S5	1.65
29.2.1.1	protein.synthesis.ribosomal protein.prokaryotic	loc_os05g45220.1	50S ribosomal protein L20	0.57
29.2.1.1	protein.synthesis.ribosomal protein.prokaryotic	loc_os01g54540.1	50S ribosomal protein L13	1.53
29.2.1.1	protein.synthesis.ribosomal protein.prokaryotic	loc_os02g57670.1	50S ribosomal protein L9	1.52
29.2.1.1	protein.synthesis.ribosomal protein.prokaryotic	loc_os01g48690.1	ribosomal protein rpS20	1.65
29.2.1.1	protein.synthesis.ribosomal protein.prokaryotic	loc_os02g09590.1	ribosome-like protein	1.52
29.2.1.1	protein.synthesis.ribosomal protein.prokaryotic	loc_os06g46930.1	50S ribosomal protein L24	1.55
29.2.1.1	protein.synthesis.ribosomal protein.prokaryotic	loc_os01g44210.1	50S ribosomal protein L31	1.71
29.2.1.1	protein.synthesis.ribosomal protein.prokaryotic	loc_os03g61260.1	50S ribosomal protein L18	1.66

29.2.1.1	protein.synthesis.ribosomal protein.prokaryotic	loc_os03g55930.1	30S ribosomal protein S9	1.96
29.2.1.1	protein.synthesis.ribosomal protein.prokaryotic	loc_os02g51790.1	50S ribosomal protein L29	1.55
29.2.1.2	protein.synthesis.ribosomal protein.eukaryotic	loc_os03g49710.1	30S ribosomal protein S13	1.78
29.2.2	protein.synthesis.misc ribososomal protein	loc_os08g44380.2	1 60S ribosomal protein L10a-1	0.66
29.2.2	protein.synthesis.misc ribososomal protein	loc_os07g33860.1	60S ribosomal protein L44	0.56
29.2.2	protein.synthesis.misc ribososomal protein	loc_os05g38520.2	60S ribosomal protein L36-2	0.63
29.2.2	protein.synthesis.misc ribososomal protein	loc_os07g08330.1	60S ribosomal protein L4	0.60
29.2.2	protein.synthesis.misc ribososomal protein	loc_os08g10608.1	40S ribosomal protein S11	0.66
29.2.2	protein.synthesis.misc ribososomal protein	loc_os03g38260.1	60S ribosomal protein L19-3	1.75
29.2.2	protein.synthesis.misc ribososomal protein	loc_os03g21940.1	60S ribosomal protein L19-3	1.51
29.2.2	protein.synthesis.misc ribososomal protein	loc_os02g40880.1	60 ribosomal protein L14	0.63
29.2.2	protein.synthesis.misc ribososomal protein	loc_os03g27260.1	40S ribosomal protein S6	0.65
29.2.2	protein.synthesis.misc ribososomal protein	loc_os03g29460.1	60S ribosomal protein L27a-3	0.13
29.2.2	protein.synthesis.misc ribososomal protein	loc_os02g56990.1	60S ribosomal protein L37	1.55
29.2.2	protein.synthesis.misc ribososomal protein	loc_os04g42270.2	60S ribosomal protein L23a	0.66
29.2.99	protein.synthesis.misc	loc_os04g56480.1	protein pelota	0.56
29.3.2	protein.targeting.mitochondria	loc_os05g44916.1	mitochondrial-processing peptidase alpha subunit	1.75
29.3.3	protein.targeting.chloroplast	loc_os10g35030.1	IAP100	2.35
29.3.3	protein.targeting.chloroplast	loc_os03g61890.1	chloroplast protein import component Toc159	1.60
29.3.4.1	protein.targeting.secretory pathway.ER	loc_os04g53220.1	signal recognition particle 14 kDa protein	0.65
29.4	protein.postranslational modification	loc_os02g10120.1	lipoxygenase 2.3	2.11
29.4	protein.postranslational modification	loc_os01g40630.1	carboxy-lyase	1.83
29.4	protein.postranslational modification	loc_os01g64970.1	serine/threonine-protein kinase SAPK4	1.55
29.4	protein.postranslational modification	loc_os05g47560.1	serine/threonine-protein kinase SNT7	1.71

29.4	protein.postranslational modification	loc_os04g59190.1	peroxidase 2 precursor	0.63
29.4.1.57	protein.postranslational modification.kinase.receptor like cytoplasmatic kinase VII	loc_os11g46880.1	wall-associated kinase 3	1.92
29.5	protein.degradation	loc_os06g11400.1	threonine endopeptidase	0.48
29.5	protein.degradation	loc_os01g47450.1	carboxyl-terminal-processing protease precursor	1.59
29.5	protein.degradation	loc_os10g28030.1	acylamino-acid-releasing enzyme	0.64
29.5	protein.degradation	loc_os06g04530.1	ATP-dependent Clp protease proteolytic subunit 2	1.53
29.5	protein.degradation	loc_os06g21380.1	carboxyl-terminal-processing protease precursor	1.84
29.5	protein.degradation	loc_os06g45360.1	peptidase C22G7.01c	0.64
29.5	protein.degradation	loc_os01g04900.1	metalloendopeptidase	0.63
29.5.1	protein.degradation.subtilases	loc_os05g35740.1	Pi starvation-induced protein	0.52
29.5.1	protein.degradation.subtilases	loc_os01g58280.1	cucumisin precursor	0.34
29.5.3	protein.degradation.cysteine protease	loc_os09g39070.1	thiol protease SEN102 precursor	0.61
29.5.3	protein.degradation.cysteine protease	loc_os03g54130.1	cysteine protease 1 precursor	1.62
29.5.3	protein.degradation.cysteine protease	loc_os04g28250.1	cysteine proteinase inhibitor B	0.48
29.5.4	protein.degradation.aspartate protease	loc_os11g08100.1	aspartic proteinase Asp1 precursor	0.49
29.5.5	protein.degradation.serine protease	loc_os01g06490.1	serine carboxypeptidase K10B2.2 precursor	0.60
29.5.5	protein.degradation.serine protease	loc_os11g14170.1	serine protease HTRA1 precursor	0.61
29.5.5	protein.degradation.serine protease	loc_os02g46260.1	serine carboxypeptidase 1 precursor	0.61
29.5.5	protein.degradation.serine protease	loc_os04g38640.1	protease Do-like 1	1.62
29.5.9	protein.degradation.AAA type	loc_os03g58790.1	ATPase 3	0.55
29.5.9	protein.degradation.AAA type	loc_os01g42030.1	mitochondrial chaperone BCS1	0.64

29.5.11.4.2	protein.degradation.ubiquitin.E3.RING	loc_os03g28990.1	protein binding protein	3.41
29.5.11.4.5.2	protein.degradation.ubiquitin.E3.BTB/POZ Cullin3.BTB/POZ	loc_os07g15490.1	TD and POZ domain-containing protein 1	1.53
29.5.11.20	protein.degradation.ubiquitin.proteasom	loc_os02g42320.2	proteasome subunit alpha type 2	0.57
29.5.11.20	protein.degradation.ubiquitin.proteasom	loc_os06g39870.1	26S protease regulatory subunit 8	1.66
29.5.11.20	protein.degradation.ubiquitin.proteasom	loc_os06g48640.1	26S proteasome non-ATPase regulatory subunit 2	0.51
29.6	protein.folding	loc_os07g09040.1	peptidyl-prolyl cis-trans isomerase, FKBP-type family protein	2.53
29.6	protein.folding	loc_os02g30320.1	drought-induced protein 1	1.53
29.6	protein.folding	loc_os03g14040.1	expressed protein	1.73
29.6	protein.folding	loc_os02g51570.1	FKBP-type peptidyl-prolyl cis-trans isomerase 4	1.73
29.6	protein.folding	loc_os06g34690.1	T-complex protein 1 subunit gamma	0.65
29.6	protein.folding	loc_os06g45340.1	FKBP-type peptidyl-prolyl cis-trans isomerase 4	1.78
29.6	protein.folding	loc_os03g50080.1	FK506 binding protein	1.55
29.8	protein.assembly and cofactor ligation	loc_os08g06530.1	rubredoxin family protein	1.54
Signalling				
30.2.3	signalling.receptor kinases.leucine rich repeat III	loc_os11g31540.1	BRASSINOSTEROID INSENSITIVE 1-associated receptor kinase 1 precursor	0.60
30.2.11	signalling.receptor kinases.leucine rich repeat XI	loc_os02g40240.1	leucine-rich repeat receptor protein kinase EXS precursor	0.58

30.2.17	signalling.receptor kinases.DUF 26	loc_os04g52614.1	SHR5-receptor-like kinase	1.79
30.2.17	signalling.receptor kinases.DUF 26	loc_os07g43570.1	CRK10	2.03
30.2.17	signalling.receptor kinases.DUF 26	loc_os07g35310.1	CRK10	0.58
30.2.17	signalling.receptor kinases.DUF 26	loc_os07g43560.1	CRK10	2.48
30.2.17	signalling.receptor kinases.DUF 26	loc_os07g35370.1	receptor-like protein kinase homolog RK20-1	0.62
30.2.17	signalling.receptor kinases.DUF 26	loc_os08g04210.1	protein kinase	0.66
30.2.17	signalling.receptor kinases.DUF 26	loc_os03g16950.1	serine/threonine kinase-like protein	0.54
30.2.99	signalling.receptor kinases.misc	loc_os04g56430.1	CRK5	0.62
30.3	signalling.calcium	loc_os01g40630.1	carboxy-lyase	1.83
30.3	signalling.calcium	loc_os03g21380.2	calcium-binding protein CAST	0.53
30.3	signalling.calcium	loc_os05g06920.1	guanosine-3,5-bis 3-pyrophosphohydrolase	1.96
30.3	signalling.calcium	loc_os01g72100.1	calcium-binding protein CML10	1.53
30.4.5	signalling.phosphinositides.inositol-1,3,4-trisphosphate 5/6-kinase	loc_os09g34300.1	inositol 1, 3, 4-trisphosphate 5/6-kinase family protein	0.61
30.5	signalling.G-proteins	loc_os03g56840.1	GTP binding protein, putative, expressed	1.73
30.5	signalling.G-proteins	loc_os12g37360.1	GTP-binding protein SAR1A	0.48
30.11	signalling.light	loc_os11g02610.1	RPT2-like protein	1.74
30.11	signalling.light	loc_os10g01044.1	tic62 protein	1.92
Cell				
31.1	cell.organisation	loc_os07g09520.1	expressed protein	0.60
31.1	cell.organisation	loc_os09g38090.1	expressed protein	1.68
31.1	cell.organisation	loc_os10g42500.1	plastid-lipid associated protein 3, chloroplast precursor	1.69
31.2	cell.division	loc_os02g34860.1	HECT domain and RCC1-like	0.59

			domain-containing protein 2	
31.3.1	cell.cycle.peptidylprolyl isomerase	loc_os08g19610.2	peptidyl-prolyl cis-trans isomerase, cyclophilin-type family protein	1.56
31.3.1	cell.cycle.peptidylprolyl isomerase	loc_os07g37830.1	peptidyl-prolyl cis-trans isomerase CYP37	1.52
31.4	cell.vesicle transport	loc_os03g57310.1	protein syntaxin 121	0.60
Development				
33.1	development.storage proteins	loc_os05g09704.1	stem 28 kDa glycoprotein precursor	0.60
33.1	development.storage proteins	loc_os05g10210.1	stem 28 kDa glycoprotein precursor	0.42
33.1	development.storage proteins	loc_os05g10310.2	acid phosphatase	0.59
33.1	development.storage proteins	loc_os09g37976.1	legumin-like protein	0.63
33.99	development.unspecified	loc_os11g44810.1	auxin-repressed 12.5 kDa protein	0.28
33.99	development.unspecified	loc_os02g42450.1	ripening-related protein 2 precursor	0.61
33.99	development.unspecified	loc_os01g51870.1	S-adenosylmethionine-dependent methyltransferase	0.55
33.99	development.unspecified	loc_os12g24020.1	senescence-associated protein DIN1	0.63
33.99	development.unspecified	loc_os06g22690.1	bundle sheath defective protein 2	1.65
33.99	development.unspecified	loc_os05g07890.1	lipase/lipooxygenase, PLAT/LH2	1.61
33.99	development.unspecified	loc_os09g25760.1	senescence-associated protein DH	0.63
33.99	development.unspecified	loc_os06g13030.1	LIM domain containing protein	0.52
33.99	development.unspecified	loc_os06g04990.1	early nodulin 93, putative	0.43
Transporter				
34.2	transporter.sugars	loc_os07g01560.1	sugar transport protein 1	0.38
34.2	transporter.sugars	loc_os12g32940.1	major myo-inositol transporter iolT	1.52
34.2	transporter.sugars	loc_os03g10090.1	polyol transporter protein 4	1.67
34.2	transporter.sugars	loc_os07g37320.1	sugar carrier protein C	0.51
34.3	transport.amino acids	loc_os02g55890.1	pyrophosphate-energized vacuolar	0.38

			membrane proton pump	
34.3	transport.amino acids	loc_os08g03350.1	LHT1	0.58
34.3	transport.amino acids	loc_os12g42850.1	high-affinity cationic amino acid transporter 1	1.56
34.3	transport.amino acids	loc_os06g42720.1	amino acid transporter-like protein	2.11
34.5	transport.ammonium	loc_os02g40730.1	ammonium transporter 1, member 2	1.65
34.6	transport.sulphate	loc_os09g06499.1	sulfate transporter 4.1, chloroplast precursor	1.55
34.6	transport.sulphate	loc_os04g55800.1	sulfate transporter 3.3	1.65
34.7	transport.phosphate	loc_os02g38020.1	inorganic phosphate transporter 2-1, chloroplast precursor	1.72
34.7	transport.phosphate	loc_os01g17240.1	sialin, putative	1.65
34.8	transport.metabolite transporters at the envelope membrane	loc_os07g33910.2	glucose-6-phosphate/phosphate translocator 2	0.526
34.8	transport.metabolite transporters at the envelope membrane	loc_os12g33080.1	2-oxoglutarate/malate translocator	1.95
34.9	transport.metabolite transporters at the mitochondrial membrane	loc_os11g24450.1	mitochondrial 2-oxoglutarate/malate carrier protein	1.71
34.9	transport.metabolite transporters at the mitochondrial membrane	loc_os06g10810.1	phosphate carrier protein	0.59
34.12	transport.metal	loc_os03g08070.1	copper-transporting ATPase PAA1	1.53
34.13	transport.peptides and oligopeptides	loc_os03g54000.1	oligopeptide transporter 3	1.58
34.15	transport.potassium	loc_os03g38260.1	60S ribosomal protein L19-3	1.75
34.16	transport.ABC transporters and multidrug resistance systems	loc_os01g50100.1	multidrug resistance protein 4	0.51
34.99	transport.misc	loc_os09g15170.1	permease	1.66

34.99	transport.misc	loc_os03g58080.1	biopterin transport-related protein BT1	1.65
34.99	transport.misc	loc_os03g53310.1	suppressor/enhancer of lin-12 protein 9 precursor	0.59
Not assigned				
35.1	not assigned.no ontology	loc_os12g25210.1	signal peptidase complex subunit 1	0.64
35.1	not assigned.no ontology	loc_os07g34950.1	permeases of the major facilitator superfamily	2.04
35.1	not assigned.no ontology	loc_os09g36240.3	n deoxyribodipyrimidine photolyase family protein	1.61
35.1	not assigned.no ontology	loc_os03g02590.3	peroxisomal membrane protein PEX11-1	1.57
35.1	not assigned.no ontology	loc_os05g51240.1	sigma factor sigB regulation protein rsbQ	1.54
35.1	not assigned.no ontology	loc_os01g63990.1	catalytic/ hydrolase	0.58
35.1	not assigned.no ontology	loc_os10g28080.1	chitinase 1 precursor	0.62
35.1	not assigned.no ontology	loc_os10g28120.1	chitinase 1 precursor	0.56
35.1	not assigned.no ontology	loc_os09g10820.1	NAD-dependent dyhydrogenase, Gfo/Idh/MocA family	0.66
35.1	not assigned.no ontology	loc_os05g48750.1	3-deoxy-manno-octulosonate cytidylyltransferase	1.57
35.1	not assigned.no ontology	loc_os06g17870.1	nitrate-induced NOI protein	1.53
35.1	not assigned.no ontology	loc_os05g43770.1	import inner membrane translocase subunit TIM50	0.57
35.1	not assigned.no ontology	loc_os03g29190.2	PDI-like protein	0.66
35.1	not assigned.no ontology	loc_os04g51080.1	scramblase family protein	1.67
35.1	not assigned.no ontology	loc_os05g03000.2	protein alx	1.75
35.1	not assigned.no ontology	loc_os01g11860.1	4-methyl-5-thiazole monophosphate biosynthesis protein	0.66

35.1	not assigned.no ontology	loc_os01g33160.1	pop3 peptide	0.64
35.1	not assigned.no ontology	loc_os07g09520.1	expressed protein	0.60
35.1	not assigned.no ontology	loc_os10g02630.1	U1 snRNP 70K protein	0.60
35.1	not assigned.no ontology	loc_os07g38850.1	prenyltransferase/ zinc ion binding protein	1.56
35.1	not assigned.no ontology	loc_os05g44380.1	MPPN domain containing protein	0.56
35.1	not assigned.no ontology	loc_os01g21560.1	monoglyceride lipase	0.61
35.1	not assigned.no ontology	loc_os05g51420.1	hypersensitive-induced response protein	0.61
35.1	not assigned.no ontology	loc_os08g37940.1	HAD-superfamily hydrolase subfamily IA	1.95
35.1	not assigned.no ontology	loc_os03g62010.1	harpin-induced protein	0.65
35.1	not assigned.no ontology	loc_os05g33280.1	beta-propeller domains of methanol dehydrogenase type	1.72
35.1	not assigned.no ontology	loc_os01g19740.1	CP12-1	2.28
35.1	not assigned.no ontology	loc_os07g39280.1	integral membrane protein like	1.56
35.1	not assigned.no ontology	loc_os05g30970.1	copine-8	1.55
35.1	not assigned.no ontology	loc_os08g34190.1	stromal cell-derived factor 2 precursor	0.64
35.1	not assigned.no ontology	loc_os03g24580.1	endoplasmic reticulum protein	0.59
35.1	not assigned.no ontology	loc_os06g03640.1	protein binding protein	2.07
35.1	not assigned.no ontology	loc_os06g10710.1	hypothetical protein	1.53
35.1	not assigned.no ontology	loc_os10g01080.1	pyridoxin biosynthesis protein ER1	1.57
35.1	not assigned.no ontology	loc_os12g26290.1	alpha-DOX2	1.74
35.1	not assigned.no ontology	loc_os01g01280.1	expressed protein	2.06
35.1	not assigned.no ontology	loc_os02g39930.1	OsTIL-1 - Oryza sativa Temperature-induced lipocalin-1	0.61
35.1	not assigned.no ontology	loc_os03g19380.1	CP12-2	1.93
35.1	not assigned.no ontology	loc_os03g36750.1	protein cbbY	1.52
35.1	not assigned.no ontology	loc_os01g47190.1	phosphoglycerate mutase family protein	1.51

35.1	not assigned.no ontology	loc_os03g26000.1	expressed protein	0.45
35.1	not assigned.no ontology	loc_os03g62490.1	mitochondrial prohibitin complex protein 2	0.54
35.1	not assigned.no ontology	loc_os01g53060.1	peroxisomal membrane protein 2	2.07
35.1	not assigned.no ontology	loc_os11g34850.1	maf-like protein CV_0124	1.65
35.1	not assigned.no ontology	loc_os03g53310.1	suppressor/enhancer of lin-12 protein 9 precursor	0.59
35.1	not assigned.no ontology	loc_os04g55180.1	catalytic/ hydrolase	1.54
35.1	not assigned.no ontology	loc_os03g04110.1	receptor-like GPI-anchored protein 2	0.55
35.1	not assigned.no ontology	loc_os02g42960.1	expressed protein	1.54
35.1	not assigned.no ontology	loc_os08g42980.1	RNA binding protein	0.60
35.1	not assigned.no ontology	loc_os05g34630.1	alpha/beta hydrolase fold	1.53
35.1	not assigned.no ontology	loc_os03g10180.1	expressed protein	1.66
35.1	not assigned.no ontology	loc_os03g29240.1	PDI-like protein	0.58
35.1	not assigned.no ontology	loc_os02g45344.1	acyltransferase	0.55
35.1	not assigned.no ontology	loc_os08g31750.1	S-adenosylmethionine-dependent methyltransferase	1.62
35.1	not assigned.no ontology	loc_os06g49160.1	thylakoid lumenal 16.5 kDa protein	1.66
35.1.1	not assigned.no ontology.ABC1 family protein	loc_os02g57160.1	ABC-1	1.87
35.1.5	not assigned.no ontology.pentatricopeptide (PPR) repeat-containing protein	loc_os10g28600.1	PGR3	1.67
35.1.5	not assigned.no ontology.pentatricopeptide (PPR) repeat-containing protein	loc_os10g32300.1	TPR Domain containing protein	1.60
35.1.5	not assigned.no ontology.pentatricopeptide (PPR) repeat-containing protein	loc_os04g55230.1	ataxin-2 C-terminal region family protein	1.61
35.1.5	not assigned.no ontology.pentatricopeptide (PPR)	loc_os03g37260.1	ATP binding protein	1.52

	repeat-containing protein			
35.1.5	not assigned.no ontology.pentatricopeptide (PPR) repeat-containing protein	loc_os03g04390.1	vegetative storage protein	1.65
35.1.5	not assigned.no ontology.pentatricopeptide (PPR) repeat-containing protein	loc_os06g06550.1	expressed protein	0.55
35.1.5	not assigned.no ontology.pentatricopeptide (PPR) repeat-containing protein	loc_os12g06650.1	tetratricopeptide-like helical	1.55
35.1.14	not assigned.no ontology.S RNA-binding domain-containing protein	loc_os03g62780.1	RNA binding S1	1.68
35.1.19	not assigned.no ontology.C2 domain-containing protein	loc_os07g31720.1	ZAC	2.69
35.1.19	not assigned.no ontology.C2 domain-containing protein	loc_os02g10480.1	GTPase activating protein	0.66
35.1.40	not assigned.no ontology.glycine rich proteins	loc_os04g10380.1	expressed protein	1.51
35.1.41	not assigned.no ontology.hydroxyproline rich proteins	loc_os11g01439.1	expressed protein	1.54
35.1.41	not assigned.no ontology.hydroxyproline rich proteins	loc_os03g21040.2	expressed protein	0.66
35.1.41	not assigned.no ontology.hydroxyproline rich proteins	loc_os07g48490.2	expressed protein	0.63
35.1.42	not assigned.no ontology.proline rich family	loc_os02g35090.1	expressed protein	1.78
35.2	not assigned.unknown	loc_os03g06230.1	expressed protein	1.58
35.2	not assigned.unknown	loc_os06g09020.1	expressed protein	2.11
35.2	not assigned.unknown	loc_os10g22460.1	protein phosphatase 2C	1.99
35.2	not assigned.unknown	loc_os02g03670.1	expressed protein	1.69
35.2	not assigned.unknown	loc_os12g25210.1	signal peptidase complex subunit 1	0.64
35.2	not assigned.unknown	loc_os07g27280.1	conserved hypothetical	0.54
35.2	not assigned.unknown	loc_os07g06990.1	expressed protein	1.52
35.2	not assigned.unknown	loc_os08g17390.1	expressed protein	1.91
35.2	not assigned.unknown	loc_os05g49060.1	expressed protein	0.56
35.2	not assigned.unknown	loc_os07g28610.1	thylakoid membrane phosphoprotein 14	1.87

			kda	
35.2	not assigned.unknown	loc_os02g03250.1	expressed protein	2.00
35.2	not assigned.unknown	loc_os05g43860.1	expressed protein	1.58
35.2	not assigned.unknown	loc_os12g27010.1	transposon protein, putative, CACTA, En/Spm sub-class	1.67
35.2	not assigned.unknown	loc_os04g13880.1	retrotransposon protein	1.87
35.2	not assigned.unknown	loc_os05g47470.2	VIP1 protein	0.57
35.2	not assigned.unknown	loc_os03g14642.1	36.4 kDa proline-rich protein	0.61
35.2	not assigned.unknown	loc_os01g73670.3	expressed protein	0.55
35.2	not assigned.unknown	loc_os01g10400.2	expressed protein	2.30
35.2	not assigned.unknown	loc_os02g13060.1	expressed protein	1.52
35.2	not assigned.unknown	loc_os04g45600.1	expressed protein	2.27
35.2	not assigned.unknown	loc_os04g21350.2	flowering promoting factor-like 1	1.70
35.2	not assigned.unknown	loc_os08g32930.1	expressed protein	2.83
35.2	not assigned.unknown	loc_os07g38840.1	expressed protein	0.50
35.2	not assigned.unknown	loc_os08g43180.1	expressed protein	0.60
35.2	not assigned.unknown	loc_os01g03360.1	Bowman-Birk type bran trypsin inhibitor precursor	0.34
35.2	not assigned.unknown	loc_os05g22614.1	expressed protein	1.92
35.2	not assigned.unknown	loc_os04g20810.3	receptor protein kinase-like protein	0.53
35.2	not assigned.unknown	loc_os03g01490.1	expressed protein	3.18
35.2	not assigned.unknown	loc_os03g49190.1	oleosin 18 kDa	0.49
35.2	not assigned.unknown	loc_os04g42800.1	expressed protein	1.61
35.2	not assigned.unknown	loc_os06g12580.1	pro-resilin precursor	0.61
35.2	not assigned.unknown	loc_os08g25700.1	expressed protein	1.59
35.2	not assigned.unknown	loc_os07g09800.1	expressed protein	1.88

35.2	not assigned.unknown	loc_os07g07540.1	SHOOT1 protein	2.11
35.2	not assigned.unknown	loc_os02g49680.1	calcium sensing receptor	2.07
35.2	not assigned.unknown	loc_os05g01530.1	expressed protein	1.78
35.2	not assigned.unknown	loc_os03g64020.1	expressed protein	1.78
35.2	not assigned.unknown	loc_os01g07090.1	protein phosphatase 2C homolog 7	1.69
35.2	not assigned.unknown	loc_os05g33520.1	expressed protein	2.36
35.2	not assigned.unknown	loc_os11g34870.1	tubulin alpha-6 chain	1.88
35.2	not assigned.unknown	loc_os06g15400.1	threonine endopeptidase	1.57
35.2	not assigned.unknown	loc_os12g36850.1	major pollen allergen Bet v 1-D/H	0.40
35.2	not assigned.unknown	loc_os04g52479.1	expressed protein	2.08
35.2	not assigned.unknown	loc_os03g50530.1	3-deoxy-D-arabino-heptulosonate 7-phosphate	1.57
35.2	not assigned.unknown	loc_os01g40310.1	expressed protein	170
35.2	not assigned.unknown	loc_os04g54320.1	expressed protein	2.33
35.2	not assigned.unknown	loc_os01g53140.1	expressed protein	0.66
35.2	not assigned.unknown	loc_os06g08990.1	expressed protein	3.14
35.2	not assigned.unknown	loc_os12g36220.1	MPI, putative	0.29
35.2	not assigned.unknown	loc_os08g41460.2	expressed protein	1.62
35.2	not assigned.unknown	loc_os03g54200.1	expressed protein	1.55
35.2	not assigned.unknown	loc_os08g27010.1	APE1	1.72
35.2	not assigned.unknown	loc_os12g36830.1	pathogenesis-related protein 10	0.51
35.2	not assigned.unknown	loc_os08g05970.1	expressed protein	0.28
35.2	not assigned.unknown	loc_os02g20490.1	expressed protein	1.68
35.2	not assigned.unknown	loc_os02g49870.1	expressed protein	1.58
35.2	not assigned.unknown	loc_os02g56030.1	expressed protein	1.56
35.2	not assigned.unknown	loc_os05g33300.1	tat pathway signal sequence family protein	1.52

35.2	not assigned.unknown	loc_os02g33820.1	abscisic stress ripening protein 1	0.62
35.2	not assigned.unknown	loc_os01g68450.1	expressed protein	1.73
35.2	not assigned.unknown	loc_os02g31230.2	expressed protein	1.78
35.2	not assigned.unknown	loc_os12g23180.1	RNA binding protein	1.84
35.2	not assigned.unknown	loc_os07g09520.1	expressed protein	0.60
35.2	not assigned.unknown	loc_os01g40630.1	carboxy-lyase	1.83
35.2	not assigned.unknown	loc_os11g42490.1	expressed protein	1.68
35.2	not assigned.unknown	loc_os08g02210.1	expressed protein	1.57
35.2	not assigned.unknown	loc_os01g47630.1	expressed protein	1.71
35.2	not assigned.unknown	loc_os02g28580.1	expressed protein	1.58
35.2	not assigned.unknown	loc_os03g03470.3	expressed protein	0.37
35.2	not assigned.unknown	loc_os08g25250.1	conserved hypothetical protein	3.66
35.2	not assigned.unknown	loc_os01g70400.1	expressed protein	3.18
35.2	not assigned.unknown	loc_os06g14050.1	MAC/Perforin domain containing protein	0.36
35.2	not assigned.unknown	loc_os12g02660.2	expressed protein	2.73
35.2	not assigned.unknown	loc_os03g52700.1	expressed protein	2.73
35.2	not assigned.unknown	loc_os06g39912.1	expressed protein	1.77
35.2	not assigned.unknown	loc_os03g61090.1	expressed protein	2.35
35.2	not assigned.unknown	loc_os04g51960.1	expressed protein	0.51
35.2	not assigned.unknown	loc_os05g27780.1	expressed protein	0.57
35.2	not assigned.unknown	loc_os08g05960.1	expressed protein	0.43
35.2	not assigned.unknown	loc_os02g57020.1	expressed protein	1.82
35.2	not assigned.unknown	loc_os04g54620.1	expressed protein	0.51
35.2	not assigned.unknown	loc_os12g34920.1	csAtPR5	1.72
35.2	not assigned.unknown	loc_os05g09580.1	expressed protein	0.43
35.2	not assigned.unknown	loc_os01g59080.1	expressed protein	2.00

35.2	not assigned.unknown	loc_os03g59300.1	expressed protein	0.61
35.2	not assigned.unknown	loc_os12g18729.1	expressed protein	1.61
35.2	not assigned.unknown	loc_os03g07380.1	expressed protein	1.65
35.2	not assigned.unknown	loc_os09g36130.1	expressed protein	1.75
35.2	not assigned.unknown	loc_os01g60830.1	expressed protein	2.19
35.2	not assigned.unknown	loc_os10g32680.1	expressed protein	0.43
35.2	not assigned.unknown	loc_os12g36880.1	major pollen allergen Bet v 1-D/H	0.57
35.2	not assigned.unknown	loc_os01g50930.1	expressed protein	1.67
35.2	not assigned.unknown	loc_os01g67120.1	rhodanese-like domain containing protein	1.73
35.2	not assigned.unknown	loc_os02g51020.1	expressed protein	1.80
35.2	not assigned.unknown	loc_os08g44400.1	glutathione S-transferase, N-terminal domain containing protein	1.72
35.2	not assigned.unknown	loc_os03g06720.1	expressed protein	1.57
35.2	not assigned.unknown	loc_os05g03150.1	expressed protein	0.64
35.2	not assigned.unknown	loc_os09g29080.1	threonine endopeptidase	1.59
35.2	not assigned.unknown	loc_os01g03330.1	Bowman-Birk type bran trypsin inhibitor precursor	0.50
35.2	not assigned.unknown	loc_os11g14990.1	expressed protein	0.52
35.2	not assigned.unknown	loc_os07g41050.1	VEP1	0.66
35.2	not assigned.unknown	loc_os05g45030.1	calcium homeostasis regulator CHoR1	1.66
35.2	not assigned.unknown	loc_os03g26820.1	xylogen protein 1	0.57
35.2	not assigned.unknown	loc_os03g51950.1	expressed protein	0.65
35.2	not assigned.unknown	loc_os02g11040.1	expressed protein	0.63
35.2	not assigned.unknown	loc_os03g64030.1	receptor protein kinase-like protein	0.62
35.2	not assigned.unknown	loc_os09g34140.1	expressed protein	1.84
35.2	not assigned.unknown	loc_os05g51754.1	expressed protein	0.52

35.2	not assigned.unknown	loc_os03g49630.1	expressed protein	1.62
35.2	not assigned.unknown	loc_os05g42280.1	expressed protein	2.51
35.2	not assigned.unknown	loc_os03g29730.1	expressed protein	1.53
35.2	not assigned.unknown	loc_os12g38640.1	expressed protein	1.80
35.2	not assigned.unknown	loc_os11g37560.1	expressed protein	1.54
35.2	not assigned.unknown	loc_os03g59320.1	expressed protein	1.84
35.2	not assigned.unknown	loc_os03g30092.1	expressed protein	1.62
35.2	not assigned.unknown	loc_os06g33930.1	uncharacterized BCR, COG1963 family protein	1.52
35.2	not assigned.unknown	loc_os11g05290.1	pop3 peptide	0.63
35.2	not assigned.unknown	loc_os04g54380.1	conserved hypothetical protein	1.82
35.2	not assigned.unknown	loc_os02g48480.1	expressed protein	1.60
35.2	not assigned.unknown	loc_os05g01950.1	expressed protein	1.59

Note: [a] means specific code given by MapMan software for specific protein

[b] means protein classification assigned by MapMan software

[c] means the fold change of Dular-OE/Dular-WT

Table S2: Identification results of transcription factor by LC-MS /MS

AN[a]	Description	MP[b]	MW[c] [kDa] /PI[d]	Score
LOC_Os06g51220.4	HMG1	2	17.1/5.95	12.18
LOC_Os01g15600.1	PUR ALPHA-1	4	33.3/6.06	12.54
LOC_Os11g38900.1	histone-lysine N-methyltransferase, H3 lysine-9 specific SUVH1	5	89.2/8.54	10.93
LOC_Os02g10080.1	zinc finger C-x8-C-x5-C-x3-H type family protein	4	31.1/9.48	7.86
LOC_Os12g37720.1	zinc knuckle family protein	4	128.4/7.46	8.52
LOC_Os03g55164.1	WRKY4	2	109/7.15	2.83

Note:AN[a]：Protein accession number

MP[b]：Protein matching peptides

MW[c]：Protein relative molecular mass

PI[d]：Protein isoelectric point

Table S3: The Promo database prediction of transcription factor interaction with the *Lsi1* gene promoter.

SN[a]	TFN[b]	IP[c]	TP[d]	BS[e]
PUR alpha	T05167	145	154	CTCTGCTCCC
PUR alpha	T05167	490	499	TAGTACTCCC
HMG I(Y)	T02368	90	96	ATTTTCC
HMG I(Y)	T02368	176	182	GGAAGAA
HMG I(Y)	T02368	293	299	GAGTTCC
HMG I(Y)	T02368	430	436	GTTTTCC
HMG I(Y)	T02368	586	592	ATTTTCC
HMG I(Y)	T02368	1260	1266	GGAAAAC

SN[a]:sequence name

TFN[b]:transcription factor numbering

IP[c]:initial position

TP[d]:termination position

BS[e]:binding site

Table S4: The JASPAR database prediction of transcription factor interaction with the Lsi1 gene promoter.

SN[a]	TFN[b]	IP[c]	TP[d]	BS[e]
HMG-1	MA0044.1	1894	1902	CTTGTTCTT

SN[a]:sequence name

TFN[b]:transcription factor numbering

IP[c]:initial position

TP[d]:termination position

BS[e]:binding site

Table S5: The resulting intersection of three transcription factors

PN[a]	AN[b]	DNA pull down	PROMO	JASPAR
HMG1	LOC_Os06g51220.4	+	+	+
PURA1	LOC_Os01g15600.1	+	+	
SUVH1	LOC_Os11g38900.1	+	-	-
ZF-C3H	LOC_Os02g10080.1	+	-	-
ZFP	LOC_Os12g37720.1	+	-	-
WRKY4	LOC_Os03g55164.1	+	-	-

PN[a]:protein name

AN[b]：Protein accession number

Table S6: LC-MS/MS identification of client proteins of NIP by Co-IP

BinCode[a]	BinName[b]	ID	Description
PS			
1.1.1.2	PS.lightreaction.photosystem II.PSII polypeptide subunits	loc_os04g16770.1	photosystem Q
1.1.1.2	PS.lightreaction.photosystem II.PSII polypeptide subunits	loc_os01g31690.1	oxygen-evolving enhancer protein 1
1.1.4	PS.lightreaction.ATP synthase	loc_os09g08910.1	ATP synthase alpha subunit
1.1.4	PS.lightreaction.ATP synthase	loc_os01g49190.1	ATP synthase beta subunit
1.1.4	PS.lightreaction.ATP synthase	loc_os04g16740.1	ATP synthase alpha subunit
1.1.4	PS.lightreaction.ATP synthase	loc_os06g45120.1	vacuolar ATP synthase catalytic subunit A
1.1.5.3	PS.lightreaction.other electron carrier (ox/red).ferredoxin reductase	loc_os02g01340.1	ferredoxin-NADP reductase
1.2.5	PS.photorespiration.serine hydroxymethyltransferase	loc_os03g52840.1	serine hydroxymethyltransferase
1.2.5	PS.photorespiration.serine hydroxymethyltransferase	loc_os12g22030.1	serine hydroxymethyltransferase
1.3.1	PS.calvin cyle.rubisco large subunit	loc_os12g10580.1	ribulose bisphosphate carboxylase large chain precursor
1.3.2	PS.calvin cyle.rubisco small subunit	loc_os12g17600.1	ribulose bisphosphate carboxylase small chain C
1.3.4	PS.calvin cyle.GAP	loc_os08g34210.1	NADP-dependent glyceraldehyde-3-phosphate dehydrogenase
1.3.4	PS.calvin cyle.GAP	loc_os03g03720.1	glyceraldehyde-3-phosphate dehydrogenase B
1.3.4	PS.calvin cyle.GAP	loc_os04g38600.1	glyceraldehyde-3-phosphate dehydrogenase A
1.3.6	PS.calvin cyle.aldolase	loc_os11g07020.1	fructose-bisphosphate aldolase
1.3.6	PS.calvin cyle.aldolase	loc_os06g40640.1	fructose-bisphosphate aldolase

1.3.7	PS.calvin cyle.FBPase	loc_os03g16050.1	fructose-1,6-bisphosphatase
1.3.8	PS.calvin cyle.transketolase	loc_os06g04270.1	transketolase, chloroplast precursor
1.3.9	PS.calvin cyle.seduheptulose bisphosphatase	loc_os04g16680.1	sedoheptulose-1,7-bisphosphatase
1.3.13	PS.calvin cyle.rubisco interacting	loc_os11g47970.1	ribulose bisphosphate carboxylase/oxygenase activase
Major CHO metabolism			
2.1.1.3	major CHO metabolism.synthesis.sucrose.FBPase	loc_os01g64660.1	fructose-1,6-bisphosphatase
2.1.2.1	major CHO metabolism.synthesis.starch.AGPase	loc_os03g52460.1	glucose-1-phosphate adenylyltransferase large subunit 3
2.2.1.1	major CHO metabolism.degradation.sucrose.fructokinase	loc_os08g02120.1	fructokinase-2
2.2.1.5	major CHO metabolism.degradation.sucrose.Susy	loc_os06g09450.4	sucrose synthase 1
2.2.1.5	major CHO metabolism.degradation.sucrose.Susy	loc_os03g28330.4	sucrose synthase 2
2.2.2.1	major CHO metabolism.degradation.starch.starch cleavage	loc_os06g46340.1	alpha-glucosidase precursor
Minor CHO metabolism			
3.4.3	minor CHO metabolism.myo-inositol.InsP Synthases	loc_os03g09250.1	inositol-3-phosphate synthase
Glycolysis			
4.1	glycolysis.UGPase	loc_os09g38030.1	UTP--glucose-1-phosphate uridylyltransferase
4.2	glycolysis.PGM	loc_os03g50480.1	phosphoglucomutase
4.7	glycolysis.aldolase	loc_os06g40640.1	fructose-bisphosphate aldolase
4.7	glycolysis.aldolase	loc_os01g67860.1	fructose-bisphosphate aldolase cytoplasmic isozyme
4.8	glycolysis.TPI	loc_os01g05490.1	triosephosphate isomerase
4.8	glycolysis.TPI	loc_os09g36450.1	triosephosphate isomerase
4.9	glycolysis.glyceraldehyde 3-phosphate dehydrogenase	loc_os04g38600.1	glyceraldehyde-3-phosphate dehydrogenase A
4.9	glycolysis.glyceraldehyde 3-phosphate dehydrogenase	loc_os03g03720.1	glyceraldehyde-3-phosphate dehydrogenase B
4.11	glycolysis.phosphoglycerate mutase	loc_os01g60190.2	2,3-bisphosphoglycerate-independent phosphoglycerate mutase
4.12	glycolysis.enolase	loc_os10g08550.1	enolase

4.12	glycolysis.enolase	loc_os06g04510.1	enolase 1
Fermentation			
5.1	fermentation.LDH	loc_os09g26880.1	aldehyde dehydrogenase family 7 member A1
5.10	fermentation.aldehyde dehydrogenase	loc_os06g15990.1	aldehyde dehydrogenase
OPP			
7.1.3	OPP.oxidative PP.6-phosphogluconate dehydrogenase	loc_os06g02144.2	6-phosphogluconate dehydrogenase
7.2.2	OPP.non-reductive PP.transaldolase	loc_os01g70170.1	transaldolase 2
TCA			
8.1.1.2	TCA / org. transformation.TCA.pyruvate DH.E2	loc_os06g01630.1	dihydrolipoyllysine-residue acetyltransferase component of pyruvatedehydrogenase complex
8.1.1.3	TCA / org. transformation.TCA.pyruvate DH.E3	loc_os05g06750.1	dihydrolipoyl dehydrogenase
8.1.1.3	TCA / org. transformation.TCA.pyruvate DH.E3	loc_os01g22520.1	dihydrolipoyl dehydrogenase
8.1.1.3	TCA / org. transformation.TCA.pyruvate DH.E3	loc_os01g23610.1	dihydrolipoyl dehydrogenase
8.1.2	TCA / org. transformation.TCA.CS	loc_os02g10070.2	citrate synthase 4
8.1.3	TCA / org. transformation.TCA.aconitase	loc_os08g09200.1	aconitate hydratase
8.1.3	TCA / org. transformation.TCA.aconitase	loc_os02g03260.1	3-isopropylmalate dehydratase large subunit 2
8.1.4	TCA / org. transformation.TCA.IDH	loc_os01g46610.1	isocitrate dehydrogenase
8.2.9	TCA / org. transformation.other organic acid transformaitons.cyt MDH	loc_os10g33800.1	malate dehydrogenase
8.2.10	TCA / org. transformation.other organic acid transformaitons.malic	loc_os01g52500.1	NADP-dependent malic enzyme
8.2.11	TCA / org. transformation.other organic acid transformaitons.atp-citrate lyase	loc_os01g19450.1	ATP-citrate synthase subunit 1
8.3	TCA / org. transformation.carbonic anhydrases	loc_os01g45274.1	carbonic anhydrase

Cell wall			
10.6.2	cell wall.degradation.mannan-xylose-arabinose-fucose	loc_os11g18730.1	beta-D-xylosidase
N-metabolism			
12.2.1	N-metabolism.ammonia metabolism.glutamate synthase	loc_os07g46460.1	ferredoxin-dependent glutamate synthase
12.2.2	N-metabolism.ammonia metabolism.glutamine synthase	loc_os04g56400.1	glutamine synthetase
12.2.2	N-metabolism.ammonia metabolism.glutamine synthase	loc_os03g12290.1	glutamine synthetase root isozyme 5
12.3.1	N-metabolism.N-degradation.glutamate dehydrogenase	loc_os03g58040.1	glutamate dehydrogenase
Amino acid metabolism			
13.1.1.3.1	amino acid metabolism.synthesis.central amino acid metabolism.alanine.alanine aminotransferase	loc_os10g25130.1	alanine aminotransferase 2
13.1.1.3.1	amino acid metabolism.synthesis.central amino acid metabolism.alanine.alanine aminotransferase	loc_os07g01760.1	alanine aminotransferase 2
13.1.3.4	amino acid metabolism.synthesis.aspartate family.methionine	loc_os12g42876.1	5-methyltetrahydropteroyltriglutamate--homocysteine methyltransferase
13.1.3.4	amino acid metabolism.synthesis.aspartate family.methionine	loc_os12g42884.1	5-methyltetrahydropteroyltriglutamate--homocysteine methyltransferase
13.1.4.1	amino acid metabolism.synthesis.branched chain group.common	loc_os05g49800.1	ketol-acid reductoisomerase
13.1.4.1	amino acid metabolism.synthesis.branched chain group.common	loc_os01g32080.1	2-hydroxyphytanoyl-CoA lyase
13.1.5.3.1	amino acid metabolism.synthesis.serine-glycine-cysteine group.cysteine.OASTL	loc_os01g74650.1	cysteine synthase
13.2.3.4	amino acid metabolism.degradation.aspartate family.methionine	loc_os11g26850.2	adenosylhomocysteinase
13.2.4.3	amino acid	loc_os07g09060.1	methylmalonate-semialdehyde dehydrogenase

	metabolism.degradation.branched-chain group.valine		
13.2.5.2	amino acid metabolism.degradation.serine-glycine-cysteine group.glycine	loc_os06g40940.1	glycine dehydrogenase 2
Metal handling			
15	metal handling	loc_os01g68770.1	selenium-binding protein
Secondary metabolism			
16.2.1.1	secondary metabolism.phenylpropanoids.lignin biosynthesis.PAL	loc_os02g41630.2	phenylalanine ammonia-lyase
tetrapyrrole synthesis			
19.1	tetrapyrrole synthesis.glu-tRNA synthetase	loc_os11g34210.2	aspartyl/glutamyl-tRNA amidotransferase subunit B
19.3	tetrapyrrole synthesis.GSA	loc_os08g41990.1	glutamate-1-semialdehyde 2,1-aminomutase
Stress			
20.2.1	stress.abiotic.heat	loc_os06g50300.1	endoplasmin homolog precursor
20.2.1	stress.abiotic.heat	loc_os03g16860.1	heat shock cognate 70 kDa protein 2
20.2.1	stress.abiotic.heat	loc_os09g29840.1	endoplasmin precursor
20.2.1	stress.abiotic.heat	loc_os12g14070.1	stromal 70 kDa heat shock-related protein
20.2.1	stress.abiotic.heat	loc_os05g23740.1	stromal 70 kDa heat shock-related protein
20.2.1	stress.abiotic.heat	loc_os02g53420.1	heat shock 70 kDa protein
20.2.1	stress.abiotic.heat	loc_os02g02410.1	luminal-binding protein 3 precursor
20.2.1	stress.abiotic.heat	loc_os09g30418.1	heat shock protein 81-3
20.2.1	stress.abiotic.heat	loc_os11g47760.1	heat shock cognate 70 kDa protein 2
Redox			
21.1	redox.thioredoxin	loc_os11g09280.2	OsPDIL1-1 - Oryza sativa protein disulfide isomerase
21.2.2	redox.ascorbate and glutathione.glutathione	loc_os01g23610.1	dihydrolipoyl dehydrogenase
21.2.2	redox.ascorbate and glutathione.glutathione	loc_os07g27790.1	glutamate--cysteine ligase
21.6	redox.dismutases and catalases	loc_os03g03910.1	catalase-1
21.6	redox.dismutases and catalases	loc_os02g02400.1	catalase isozyme A
C1-metabolism			
25	C1-metabolism	loc_os09g27420.1	formate--tetrahydrofolate ligase
25	C1-metabolism	loc_os03g60090.1	methylenetetrahydrofolate reductase
Misc			
26.3	misc.gluco-, galacto- and mannosidases	loc_os11g32260.1	lysosomal alpha-mannosidase precursor
26.3	misc.gluco-, galacto- and mannosidases	loc_os06g46340.1	alpha-glucosidase precursor
RNA			
27.1	RNA.processing	loc_os07g07310.1	polynucleotide phosphorylase

DNA			
28.1.3	DNA.synthesis/chromatin structure.histone	loc_os07g36500.1	histone H4
Protein			
29.1.6	protein.aa activation.lysine-tRNA ligase	loc_os03g38980.1	lysyl-tRNA synthetase
29.1.7	protein.aa activation.alanine-tRNA ligase	loc_os10g10244.1	alanyl-tRNA synthetase
29.1.14	protein.aa activation.glycine-tRNA ligase	loc_os04g32650.1	glycyl-tRNA synthetase 1
29.1.22	protein.aa activation.asparagine-tRNA ligase	loc_os01g27520.1	asparaginyl-tRNA synthetase
29.1.40	protein.aa activation.bifunctional aminoacyl-tRNA synthetase	loc_os12g25710.1	bifunctional aminoacyl-tRNA synthetase
29.2.1.1	protein.synthesis.ribosomal protein.prokaryotic	loc_os03g20100.1	30S ribosomal protein S1
29.2.1.1	protein.synthesis.ribosomal protein.prokaryotic	loc_os12g38000.1	60S ribosomal protein L2
29.2.2	protein.synthesis.misc ribososomal protein	loc_os11g06750.1	60S ribosomal protein L3
29.2.2	protein.synthesis.misc ribososomal protein	loc_os11g04070.1	60S acidic ribosomal protein P0
29.2.2	protein.synthesis.misc ribososomal protein	loc_os01g25610.1	40S ribosomal protein S4
29.2.2	protein.synthesis.misc ribososomal protein	loc_os07g08330.1	60S ribosomal protein L4
29.2.2	protein.synthesis.misc ribososomal protein	loc_os07g41750.1	40S ribosomal protein S3-A
29.2.2	protein.synthesis.misc ribososomal protein	loc_os02g37862.1	60S ribosomal protein L6
29.2.2	protein.synthesis.misc ribososomal protein	loc_os04g28180.1	40S ribosomal protein S8
29.2.2	protein.synthesis.misc ribososomal protein	loc_os03g08440.1	40S ribosomal protein SA
29.2.2	protein.synthesis.misc ribososomal protein	loc_os03g05980.1	40S ribosomal protein S9
29.2.3	protein.synthesis.initiation	loc_os01g36920.2	spliceosome RNA helicase BAT1
29.2.3	protein.synthesis.initiation	loc_os02g05330.1	eukaryotic initiation factor 4A
29.2.4	protein.synthesis.elongation	loc_os12g35630.1	elongation factor TS family protein
29.2.4	protein.synthesis.elongation	loc_os03g08020.1	elongation factor 1-alpha
29.2.4	protein.synthesis.elongation	loc_os04g45490.1	elongation factor G
29.2.4	protein.synthesis.elongation	loc_os06g40600.1	U5 small nuclear ribonucleoprotein component
29.2.4	protein.synthesis.elongation	loc_os06g37440.1	elongation factor 1-gamma 3

29.2.4	protein.synthesis.elongation	loc_os02g32030.1	elongation factor 2
29.2.4	protein.synthesis.elongation	loc_os02g38210.1	elongation factor Tu
29.4	protein.postranslational modification	loc_os09g07510.2	serine/threonine-protein phosphatase 2A 65 kDa regulatory subunit Abeta isoform
29.4	protein.postranslational modification	loc_os07g38730.1	tubulin alpha-1 chain
29.5	protein.degradation	loc_os02g55140.1	leucine aminopeptidase 3
29.5	protein.degradation	loc_os08g44860.1	aminopeptidase N
29.5	protein.degradation	loc_os08g44860.2	aminopeptidase N
29.5.1	protein.degradation.subtilases	loc_os01g64860.1	subtilisin-like protease precursor
29.5.4	protein.degradation.aspartate protease	loc_os12g13390.1	aspartyl aminopeptidase
29.5.9	protein.degradation.AAA type	loc_os03g05730.1	cell division control protein 48 homolog E
29.5.11.20	protein.degradation.ubiquitin.proteasom	loc_os03g05730.1	cell division control protein 48 homolog E
29.6	protein.folding	loc_os06g02380.2	ruBisCO large subunit-binding protein subunit beta
29.6	protein.folding	loc_os12g17910.1	ruBisCO large subunit-binding protein subunit alpha
Signalling			
30.3	signalling.calcium	loc_os07g48100.1	CBL interacting protein kinase
30.3	signalling.calcium	loc_os08g02420.1	calmodulin
30.5	signalling.G-proteins	loc_os05g49890.1	GTP-binding nuclear protein Ran-A1
30.7	signalling.14-3-3 proteins	loc_os02g36974.4	14-3-3-like protein B
30.7	signalling.14-3-3 proteins	loc_os08g33370.2	14-3-3-like protein C
30.7	signalling.14-3-3 proteins	loc_os03g50290.1	14-3-3-like protein F
Cell			
31.1	cell.organisation	loc_os03g56810.1	tubulin beta-7 chain
31.1	cell.organisation	loc_os07g38730.1	tubulin alpha-1 chain
31.2	cell.division	loc_os03g05730.1	cell division control protein 48 homolog E
31.2	cell.division	loc_os04g35570.1	HECT domain and RCC1-like domain-containing protein 2
31.3.1	cell.cycle.peptidylprolyl isomerase	loc_os02g02890.1	peptidyl-prolyl cis-trans isomerase
Transport			
34.1	transport.p- and v-ATPases	loc_os01g51380.1	vacuolar ATP synthase subunit B isoform 1
34.1.1	transport.p- and v-ATPases.H+-transporting two-sector ATPase	loc_os06g45120.1	vacuolar ATP synthase catalytic subunit A
Not assigned			
35.1	not assigned.no ontology	loc_os11g04350.1	cell death associated protein
35.1	not assigned.no ontology	loc_os07g44410.1	WD40-like Beta Propeller Repeat family protein
35.1	not assigned.no ontology	loc_os08g02230.1	FAD binding domain containing protein
35.2	not assigned.unknown	loc_os10g30870.1	expressed protein

35.2	not assigned.unknown	loc_os03g19410.1	secreted protein
35.2	not assigned.unknown	loc_os05g45030.1	calcium homeostasis regulator CHoR1

Note：BinCode: The specific code for a specific protein given by the MapMan software

BinName: The protein analysis by MapMan software

Table S7: Primers used for protein subcellular localization, promoter clone, qPCR and BiFC

Gene	Forward sequence（5'-3'）	Reverse sequence（5'-3'）
**protein subcellular localization**		
Lsi1-Fusion	GGGGACAAGTTTGTACAAAAAAGCAG<br>GCTTCATGGCCAGCAACAACTCGAG	GGGGACCACTTTGTACAAGAAAGCTG<br>GGTCCACTTGGATGTTCTCCATCT
**promoter clone**		
Lsi1-promoter	ATGATTACGAATTCGAGCTCGCCTGAG<br>ATTTTCACCAAGTCG	TGTAGTCCATACTAGTTTCTGACGCTC<br>TATCTAGCTGAGC
**qPCR**		
HMG1	CAAGTTGGCTGTGAAGAGTAAG	CAGCGACAGATTTATTCTTGGG
PURA1	CGATATTGGGGAGAACAAGAGA	GCCGGAACAATGATTGTACTAC
SUVH1	AGCCACTTCAACTACACAACTA	GCATGTACAGGAATCGTTACAC
ZF-C3H	CATTTCAAACCGGTGTAGGAAG	AGATTGGTCATCTTTGCAACAG
ZFP	ATCACTCATATGCGCAAAGTTC	TGTCAATGGTGGAATGGGATTA
WRKY4	CCGTATGGGAGTGATTTTGATC	GAACTGAGGTGCCAAGCTAATC
Lsi1	GGGCGAACTACTCCAACGA	ACCACCTCCGACACGACCTT
ATP-α	GGCGATGGGTTGATGATAC	CTACGGAGGATGCTCTTTGA
ATP-β	CTGGGTGAGGACCACTACAA	GAAGGGCTGGCTAAGGAA
**BiFC**		
ATP-α	ATGGCGCGCCACTAGTATGGCAACCCT<br>TCGAGTCGACGA	CACCTCCTCCACTAGTAAGGGAAAACC<br>GTTCGAGTTGTTCC
ATP-β	ATGGCGCGCCACTAGTATGGCGACTC<br>GCCGGGCCCTCT	CACCTCCTCCACTAGTTGAAGCCGACTC<br>CTTGGCGATC
14-3-3f	TTTTGCCACTAGTATGTCGCCTGCTGAG<br>GCATCGCGT	TTTTCCACTAGTGTGGCCCTCTCCTTCA<br>GGCTTCGCT
CIPK	ATGGCGCGCCACTAGTATGGCGGAGCA<br>GAGAGGAAATATG	CACCTCCTCCACTAGTGCACGTTGGCTG<br>CTGCTGCTGCTTC
**GST pulldown**		
pet-32a-*Ls*<br>*i1*	GCCATGGCTGATATCGGATCCATGGCCA<br>GCAACAACTCGAG	CTCGAGTGCGGCCGCAAGCTTTCACAC<br>TTGGATGTTCTCCATCTC
Pet-32a-*C*<br>*IPK*	GCTGATATCGGAATCCGAATTCATGGCG<br>GAGCAGAGAGGAA	GTGGTGGTGGTGGTGCTCGAGGCACGT<br>TGGCTGCTGCTG
Pgex-6p-1<br>-*14-3-3*f	TTTTGGATCCATGTCGCCTGCTGAGGC<br>ATCGCGT	TTTTTCTCGAGGTGGCCCTCTCCTTCAG<br>GCTTCGCT
